# One-Electron Boron–Carbon Triel Bonding

**DOI:** 10.1021/jacs.5c13589

**Published:** 2025-09-17

**Authors:** Paula Castro Castro, Wei-Chun Liu, François P. Gabbaï

**Affiliations:** Department of Chemistry, Texas A&M University, College Station, Texas 77843-3255, United States of America

## Abstract

Aiming to interrogate the possibility of heteronuclear B–C one-electron σ-bonding, we have prepared a naphthalene-based platform ([**5**]^+^) decorated at its peri-positions by a carbenium and a boryl unit separated by a B–C distance of 2.994(4) Å. Reduction of this cationic platform by one electron affords a radical (**5** ^**•**^) with a shortened B–C distance of 2.874(3) Å, suggesting the onset of B–C one-electron bonding. This radical, which was also characterized by EPR spectroscopy, undergoes a second reduction, affording a borate ([**5**]^−^) with a long, polar covalent B–C bond of 1.793(8) Å. These compounds have been structurally, spectroscopically, and computationally investigated, shedding light on the polarization and weakness of the B–C bond of **5** ^**•**^, which is best described as a noncovalent one-electron B···C triel bond rather than a covalent one-electron σ-bond.

## Graphical abstract

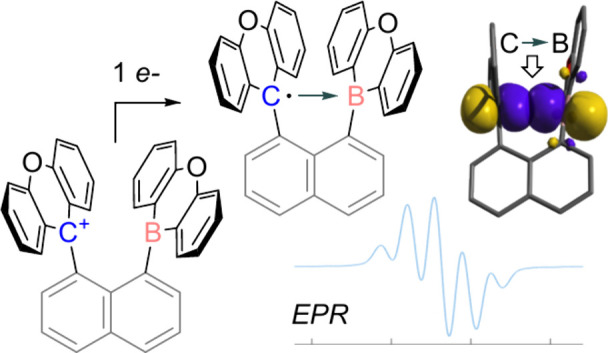

The one-electron oxidation of organoborates has emerged as a useful entry point for radical-based organic reactions. This approach has been implemented using a variety of boron substrates,^1–5^ including organotrifluoroborates,^6,7^ the oxidation of which generates radical adducts of general formula [R···BF_3_]^•^, as proposed in several working models (Figure 1).^7,8^ The methyl adduct [H_3_C···BF_3_]^•^ (**I** ^•^) is the simplest representative of such a species. While its formation can be invoked in reactions involving [CH_3_BF_3_]^−^ as a starting material,^9,10^ experimental evidence for its existence is lacking, even if a recent computational study has suggested that it exists as a noncovalent complex in which the methyl and the BF_3_ units are held by a one-electron B···C triel bond of 2.864 Å.^11^ The lack of covalency in this open-shell adduct stems from the asymmetric distribution of the unpaired density, which retains predominant carbon parentage, leading to a highly polarized and thus noncovalent one-electron bond.

**Figure 1:**
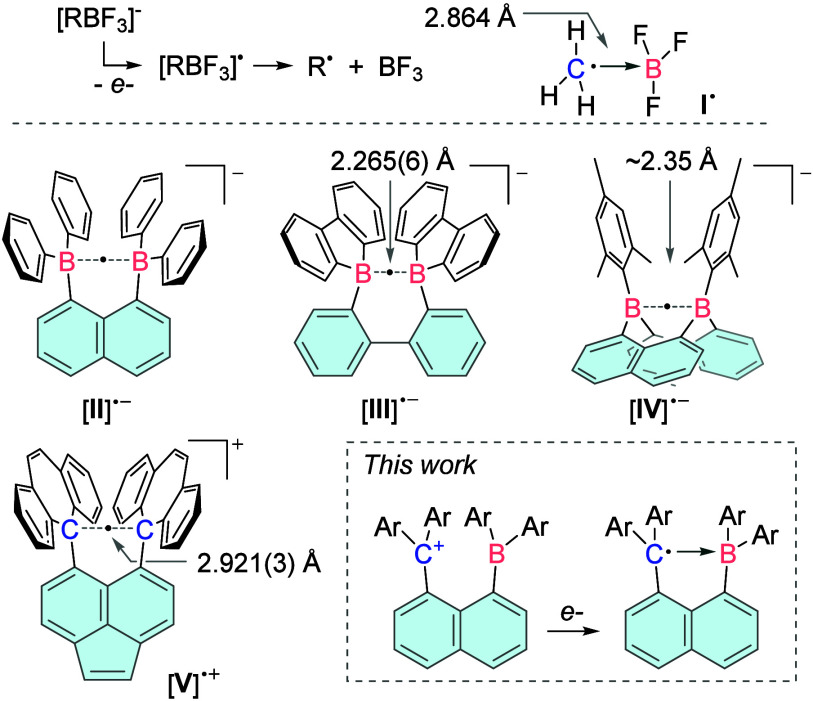
Top: Formation of [R···BF_3_]^•^ intermediates by oxidation of organotrifluoroborates and representation of the computed structure and B–C distance of [H_3_C···BF_3_]^•^. Bottom: Examples of systems featuring one-electron B–B or C–C σ-bonds, with corresponding bond lengths.

Stronger one-electron bonding interactions have been observed in homonuclear analogs of such systems. Over two decades ago, we spectroscopically characterized a diboron system ([**II**]^•–^) in which the two centers are confined within the same molecular unit (Figure 1).^12^ The existence of boron–boron half-order bonds has now been confirmed by X-ray diffraction^13^ for systems such as [**III**]^•–^ and [**IV**]^•–^ which display rather short boron–boron distances in the 2.2–2.4 Å range.^14,15^ Efforts toward the carbon analogs have also emerged, leading to the description of [**V**]^•+^ as a compound featuring a long carbon–carbon one-electron bond of 2.921(3) Å,^16^ the formulation of which has been recently disputed.^15^ Building on these developments, we have now decided to test whether the rigid linker strategy adopted in these diboron and dicarbon systems could provide access to a compound mimicking the B···C bond found in species such as **I** ^•^.^11^

Building on our past work on naphthalene systems,^12,17^ we generated carbinol **1** and converted this derivative into the xanthylium salt [**2**]­[BF_4_], providing convenient access to the methoxide-protected analog **3** (Figure 2). Lithiation of **3**, followed by reaction with 10-bromo-9-oxa-10-boraanthracene^18^ afforded compound **4**. The ^11^B­{^1^H} NMR spectrum of **4** shows a signal at 14.03 ppm, suggesting a tetracoordinate boron center. A structural assay confirmed that the methoxide group bridges^19^ the C9 position of the xanthyl unit and the boron atom, resulting in C9–O3 and B–O3 bonds of 1.484(2) Å and 1.711(2) Å, respectively (Figure 2). The methoxide unit of **4** can be cleanly removed via simple treatment with HBF_4_, to afford the xanthylium borane [**5**]^+^ as a tetrafluoroborate salt. The ^11^B­{^1^H} NMR spectrum of [**5**]­[BF_4_] displays a broad resonance at 52 ppm, close to the value measured for other oxaboranthracene derivatives.^18,20^ In the ^13^C­{^1^H} NMR spectrum, the carbenium center resonates at 176.4 ppm, consistent with the presence of a xanthylium unit.^20–23^ The crystal structure of [**5**]­[BF_4_] indicates a cofacial arrangement of the xanthylium and the oxaboraanthracene units, resulting in a C9–B separation of 2.994(4) Å (Figure 2). This distance is comparable to the B–B separation in **II** (3.002(2) Å)^12^ but slightly shorter than the C_carbenium_–C_carbenium_ separation in the 1,8-bis­(diphenylmethylium)­naphthalenediyl dication (3.112(2) Å).^17^ Both the boron and carbenium centers remain planar, as indicated by the sum of the C–B–C and C–C9–C angles of 359.8 and 359.9°, respectively.

**Figure 2:**
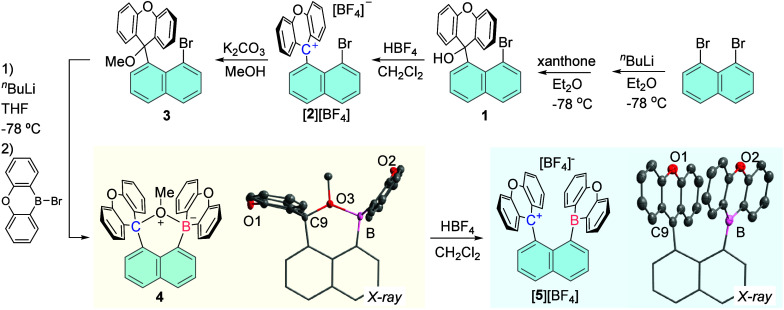
Synthetic scheme leading to the formation of [**5**]­[BF_4_]. The crystal structures of **4** and [**5**]­[BF_4_] are also shown. For all crystal structures, the ellipsoids are drawn at the 50% probability level; the solvent molecules, counterions and H atoms are omitted for clarity.

With this compound in hand and inspired by the prior characterization of open-shell B–C π-bonding,^24–30^ we became eager to test if [**5**]^+^ could support B–C one-electron bonding. Clues to this possibility were obtained by cyclic voltammetry, which shows that [**5**]­[BF_4_] undergoes two successive reductions at *E*_1/2_ values of −0.41 V and −0.81 V, respectively (vs Fc^+^/Fc) (Figure 3). These consecutive processes may indicate the sequential population of the B–C σ-orbital by one and two electrons. The observation of two successive reductions is in marked contrast to the behavior of 1,8-bis­(methylium)­naphthalene systems, which usually feature a single two-electron reduction event.^31,32^ The observation of two well-separated waves illustrates the differing nature of the two electroactive centers of [**5**]^+^ as well as the charging of the system upon injection of a second electron.

**Figure 3:**
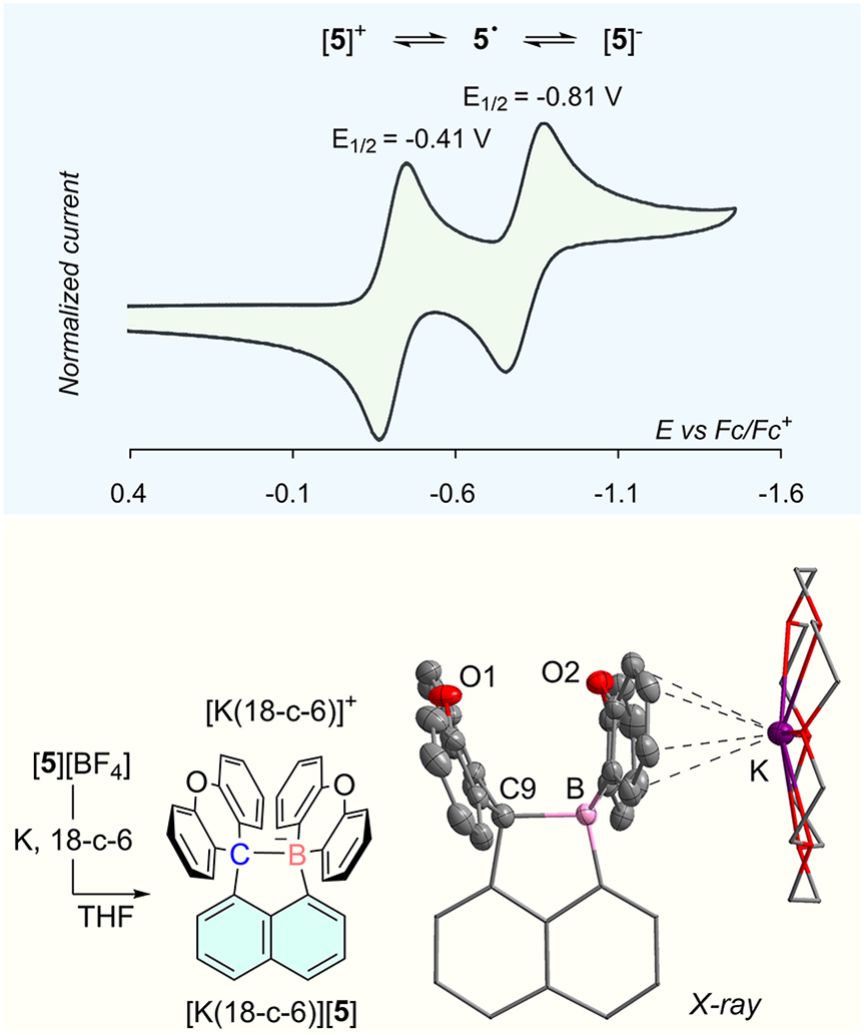
Top: Cyclic voltammogram of [**5**]­[BF_4_] recorded under N_2_ in CH_2_Cl_2_ with TBAPF_6_ as supporting electrolyte (0.25 M) at a scan rate of 0.1 V/s. Bottom: Synthesis and crystal structure of [K­(18-c-6)]­[**5**]. Ellipsoids are drawn at the 50% probability level. Solvent molecules and H atoms are omitted for clarity.

Encouraged by these results, we endeavored first to isolate the two-electron reduction product, which was easily generated by a reduction of [**5**]­[BF_4_] in THF using potassium/18-crown-6 (18-c-6). The product of this reaction, identified as [K­(18-c-6)]­[**5**], was isolated as a pale yellow powder (Figure 3). Its negative mode ESI mass spectrum shows a molecular ion at 485.1724 amu, corresponding to [**5**]^−^. The ^1^H NMR spectrum displays well-resolved aromatic resonances, generally upfield from those of [**5**]^+^ while its ^11^B­{^1^H} NMR spectrum features a single peak at −4.6 ppm, consistent with the presence of a two-electron B–C9 bond. This linkage was confirmed by a structural analysis which shows a B–C9 bond of 1.793(8) Å (Figure 3), notably shorter than the value of 2.994(4) Å found in [**5**]­[BF_4_] but longer than that measured for the B–C_sp3_ bond of [Ph_3_BMe]^−^ (1.642(16) Å).^33^ In fact, the B–C9 distance in [**5**]^−^ approaches the value of 1.89 Å recently reported for the B–B bond of a hexaaryl-substituted diboron(6) dianion^34^ or that recorded for the longest C–C bond (1.8 Å).^32^ The long B–C9 bond in [**5**]^−^, which attests to the strain imposed by the rigid naphthalene backbone and the aryl substituents, also leads to significant pyramidalization of the boron and former carbenium centers, as evinced by the sum of the C–B–C and C–C9–C angles of 333.4° and 336.8°, respectively.

Having confirmed the formation of a two-electron B–C bond in [**5**]^−^, we became eager to test whether the single-electron reduction product **5** ^**•**^ could be isolated. Toward this end, [**5**]­[BF_4_] was treated with cobaltocene (Cp_2_Co) in CH_2_Cl_2_ (Figure 4). This reaction afforded a dark red solution of **5** ^**•**^, which gave an electron paramagnetic resonance (EPR) spectrum (Figure 4) that could be modeled using hyperfine parameters in reasonable agreement with those computed (see the Supporting Information (SI)). While this EPR spectrum is similar to that of other simple xanthyl radicals,^35–37^ its fitting revealed an experimental ^11^B hyperfine parameter of 0.65 G, very close to the computed value of 0.67 G (see SI). This value is significantly lower than the 4.8–9.8 G range measured for radicals such as **II** ^•–^, **III** ^•–^, and **IV** ^•–^, pointing to the minor contribution of the boron atom to the singly occupied molecular orbital (SOMO) of **5** ^**•**^. Consistently, the spin density map of **5** ^**•**^ shows that the unpaired spin primarily occupies the π* orbital of the former xanthylium cation (Figure 4). However, an extension of the largest lobe from the C9 atom toward the boron atom shows that the unpaired spin density is polarized toward the boron atom. The formation of **5** ^**•**^ can also be observed by spectroelectrochemistry. A linear sweep from +0.4 V to −0.6 V in CH_2_Cl_2_ leads to a progressive quenching of the broad carbenium-based band at 350 and 560 nm (see SI). Concomitantly, a new feature emerges at 450 nm, which, based on TD-DFT calculations, corresponds to the radical **5** ^**•**^. As the potential becomes more cathodic, the low-energy part of the spectrum flattens, consistent with the formation of the borate [**5**]^−^.

**Figure 4:**
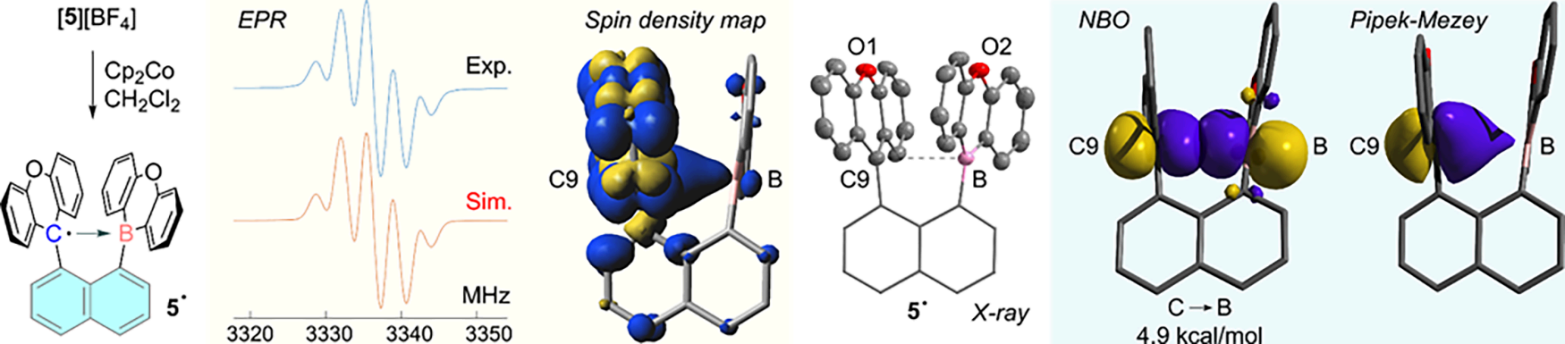
Synthesis, EPR spectrum, spin density map, and crystal structure of **5** ^•^. Inset shows NBO and Pipek-Mezey orbitals corresponding to the C···B interaction in **5** ^•^. Contour plots are drawn at an isovalue of 0.05. Ellipsoids are drawn at the 50% probability level. H atoms are omitted for clarity.

Xanthyl radical moieties such as that proposed to exist in **5** ^**•**^ have often been invoked in xanthylium reduction reactions^23,36,38–40^ and, in some instances, observed by EPR spectroscopy.^35–37^ Yet, they appear to have eluded structural characterization. Single crystals of **5** ^**•**^ were thus subjected to X-ray diffraction, leading to a structure in which the xanthyl and oxaboraanthracene units adopt an eclipsed conformation as indicated by the value of the C1–C2–C3–B dihedral angle of −1.1° (Figure 4). Comparing this value to that in [**5**]^+^ (8.4(1)°) indicates a greater alignment of the former carbenium and boron centers. This greater alignment also manifests in a shortening of the B–C9 distance from 2.994(4) Å in [**5**]­[BF_4_] to 2.874(3) Å in **5** ^**•**^. The observed shortening of 0.12 Å or 4.0% is comparable or slightly larger than that observed between **V** ^2+^ and **V** ^*•*+^ (0.11 Å or 3.6%). We also note that the B–C9 distance in **5** ^**•**^ (2.874(4) Å) is shorter than the one-electron σ bond proposed for **V** ^*•*+^ (2.921(3) Å), despite the larger size of boron (r_cov_ = 0.84 Å for B and 0.76 Å for C).^41^ These features appear consistent with the presence of a bonding interaction between the boron atom and the former carbenium center, the formation of which is supported by the rigid backbone and intramolecular π–π interactions (see SI). The central carbon and boron centers retain a trigonal planar geometry (∑(C–B–C)= 359.3° and ∑(C–C9–C) = 359.4°), signaling the absence of rehybridization that would have been expected upon the formation of a covalent one-electron σ bond. Moreover, the shortening observed upon reduction of [**5**]^+^ could be attributed to a decrease in electrostatic repulsion between the electropositive boron atom and the carbenium center. The same electrostatic argument could be advanced to explain the contraction of the central motif when **V** ^2+^ is converted into **V** ^•+^. In fact, a recently published study^15^ argues for the absence of significant C···C one-electron σ-bonding in **V** ^•+^, based on the limited contraction observed as well as several computed descriptors, including electron density (ρ­(r)) at the critical point of the C···C bond as derived from Atom in Molecules (AIM) analyses and Wiberg bond indices. We have also carried out such analyses in the case of the [**5**]^+/0/–^. The ρ­(r) value at the critical point of the B···C bond of **5** ^**•**^ (0.016 e/bohr^3^) is significantly smaller than that of [**5**]^−^ (0.116 e/bohr^3^), pointing to insignificant covalency of the central motif. The WBIs offer a corroborating picture with the value calculated for **5** ^**•**^ (0.06) significantly smaller than that of [**5**]^−^ (0.65). Given that these descriptors argue against covalent B···C one-electron σ-bonding in **5** ^•^, we propose that this one-electron interaction is best described as a supported one-electron triel bond, a situation never experimentally observed but nonetheless envisaged computationally for (*vide supra*), a weakly held single electron triel bonded complex with a calculated B···C distance of 2.864 Å,^11^ a value conspicuously close to the experimental value of 2.874(3) Å measured in **5** ^**•**^. Consistent with this description, an NBO calculation^42^ reveals the presence of a one-electron carbon-to-boron donor–acceptor interaction associated with a second-order energy of 4.9 kcal/mol (Figure 4). Additional support for the presence of this one-electron triel bonding interaction can also be gleaned from a Pipek-Mezey analysis,^43^ which shows a C-centered radical orbital extending toward the boron atom (Figure 4).

This work highlights the difficulty in achieving covalent heteronuclear one-electron σ bonding. The absence of a *bona fide* one-electron σ bond in **5** ^**•**^ reflects the elevated electron affinity of the carbenium center that significantly exceeds that of its borane counterpart. As a result, the unpaired electron resides predominantly on the carbon side of the motif, with only a minor shift of the unpaired electron density toward the boron. As a result, **5** ^**•**^ is best described as a xanthyl radical acting as a single-electron donor and thus involved in a triel bond with the neighboring boron atom. Despite its weakness, this triel bond defines a conduit along which electron density can be increased, as seen upon injection of a second electron, leading to a genuine two-electron B–C bond in [**5**]^−^. Lastly, this work might motivate the experimental pursuit of other one-electron noncovalent interactions.^44–47^

## Supplementary Material



## Notes

These materials are available free of charge via the Internet at . The Supporting Information is available free of charge at https://pubs.acs.org/doi/10.1021/jacs.5c13589.

Experimental and computational details (PDF)
