# Supplementary material for: One-Electron Boron–Carbon Triel Bonding
Source: J Am Chem Soc. 2025 Sep 17;147(39):35227–31. doi: 10.1021/jacs.5c13589 (PMC12498421; doi:10.1021/jacs.5c13589)
Supplement: Supplementary file 1 [file ja5c13589_si_001.pdf]

One-Electron Boron–Carbon Triel Bonding

Paula Castro Castro, Wei-Chun Liu, and François P. Gabbaï*

Department of Chemistry, Texas A&M University, College Station, TX 77843-3255, United States of America

email: francois@tamu.edu

Supporting Information

Table of Contents

Synthetic details S2

General Considerations S2

Crystallographic Measurements S2

Synthesis of **1** S2

Synthesis of [**2**][BF_4] S3

Synthesis of **3** S3

Synthesis of **4** S4

Synthesis of [**5**][BF_4] S5

Synthesis of $\mathbf{5}^{\bullet}$ S5

Synthesis of [K(18-c-6)][**5**] S6

NMR spectra and crystal structures S7

Cyclic Voltammetry and Differential Pulse Voltammetry S21

EPR spectroscopy S21

UV-vis spectroscopy S22

Spectroelectrochemical Analysis S23

Computational Analysis S24

References S32

Synthetic details

General Considerations

10-Bromo-9-oxa-10-boraanthracene[1] was prepared following literature methods. 1,8-Dibromonaphthalene and xanthone were purchased from Thermo Scientific Chemicals and BeanTown Chemical and used as received. Solvents were dried over Na/K (Et_2O and THF) and CaH_2 (DCM) by refluxing under N_2. All other solvents were ACS reagent grade and used as received. All compounds were synthesized under a dry N_2 atmosphere with standard Schlenk techniques or in a nitrogen-filled glove box unless otherwise specified. ^{1}H, $^{13}C\{^{1}H\}$ and $^{11}B\{^{1}H\}$ NMR spectra were recorded at room temperature on a Bruker Avance 500 NMR spectrometer or a Bruker Ascend 400 NMR spectrometer. Chemical shifts are given in ppm and are referenced to residual ^{1}H and ^{13}C solvent signals. Mass spectrometric analyses were performed in-house at the Center for Mass Spectrometry. Elemental analyses were performed at Atlantic Microlab (Norcross, GA).

Crystallographic Measurements

Crystallographic measurements were performed at 110 K using a Bruker D8 QUEST diffractometer (Mo-Kα radiation, λ = 0.71069 Å) equipped with Photon III detectors and a Rigaku XtaLAB Synergy-S diffractometer (Cu-Kα radiation, λ = 1.5418 Å) with a Dectris Eiger 2 detector. A specimen of suitable size and quality was selected and mounted onto a nylon loop in each case. Integrated intensity information for each reflection was obtained by reducing the data frames with the program Bruker AXS APEX4 and CrysAlis^{Pro} [2] The semiempirical method SADABS was used for absorption corrections.[3] The structures were solved by direct methods (ShelXT)[4] and refined by the full-matrix least-square technique against F^2 with anisotropic temperature-dependent parameters for all non-hydrogen atoms (ShelXL)[5] using the Olex2 interface.[6] All H-atoms were geometrically placed and refined using the riding atom model. Diamond 4 was employed for the final data presentation and structure plots. The data has been deposited with the Cambridge Structural Database. CCDC 2478363-247869 contains the supplementary crystallographic data for this paper.

CCDC number	Compound
2478363	**1**
2478364	[**2**][BF_4]
2478365	**3**
2478366	**4**
2478367	[**5**][BF_4]
2478368	**5**$^{\bullet}$
2478369	[K(18-c-6)][**5**]

Synthesis of 1

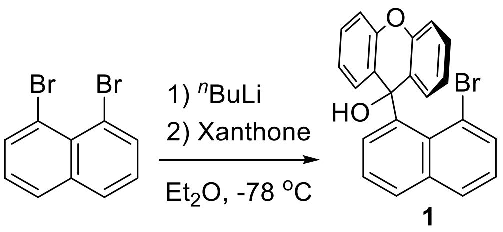

^{n}BuLi (3.2 mL, 8.00 mmol, 2.5 M in hexanes) was added to a solution of 1,8-dibromonaphthalene (2.2 g, 7.69 mmol) in Et_2O (20 mL) at -78 °C, leading to a color change from white to pale yellow. The resulting solution was stirred at that temperature for 2 hours, at which point xanthone (1.7 g, 8.67 mmol) was added as a solid. The reaction mixture was warmed to room temperature and stirred overnight, and then quenched by adding DI water (10 mL). After removing the Et_2O via rotary evaporation, the remaining aqueous phase was extracted with CH_2Cl_2 (3 × 30 mL). The organic fractions were combined, dried over $MgSO_4$, and dried under vacuum to give a pale-yellow solid, which was further purified by rinsing with hexanes and acetonitrile. This procedure afforded **1** as a white solid (2.6 g, 6.45 mmol, 84%). Single crystals suitable for

X-ray crystal structure analysis were grown by layering pentane in a CH_2Cl_2 solution of **1** under an ambient atmosphere. **^{1}H NMR** (499.41 MHz, $CDCl_3$) δ 8.92 (dd, J = 7.6, 1.3 Hz, 1H, naphthalene-H), 7.98 (dd, J = 8.1, 1.1 Hz, 1H, naphthalene-H), 7.88 (dd, J = 8.1, 1.1 Hz, 1H, naphthalene-H), 7.70 (t, J = 7.8 Hz, 1H, naphthalene-H), 7.54 (dd, J = 7.4, 1.3 Hz, 1H, naphthalene-H), 7.34 (td, J = 6.9, 1.6 Hz, 2H, xanthenol-H), 7.26 (d, J = 8.0 Hz, 2H, xanthenol-H), 7.15 (t, J = 7.8 Hz, 1H, naphthalene-H), 6.90 (td, J = 6.9, 1.1 Hz, 2H, xanthenol-H), 6.81 (dd, J = 7.8, 1.5 Hz, 2H, xanthenol-H), 2.16 (s, 1H, xanthenol-OH). **^{13}C{^{1}H} NMR** (125.76 MHz, $CDCl_3$) δ 150.31 (s, xanthenol-C), 137.62 (s, naphthalene-C), 137.34 (s, naphthalene-C), 135.21 (s, naphthalene-CH), 131.35 (s, naphthalene-CH), 130.94 (s, naphthalene-C), 130.89 (s, naphthalene-CH), 129.95 (s, naphthalene-CH), 129.43 (s, xanthenol-C), 129.39 (s, xanthenol-CH), 126.76 (s, xanthenol-CH), 125.82 (s, naphthalene-CH), 125.21 (s, naphthalene-CH), 123.07 (s, xanthenol-CH), 118.82 (s, naphthalene-C), 116.60 (s, xanthenol-CH), 72.88 (s, xanthenol-COH). ESI-MS calcd for $^{12}C_{23}{}^1H_{14}{}^{79}Br^{16}O^+$ $[M]^+$ 385.0223, found: 385.0206. The identity of this compound was further established by single-crystal X-ray diffraction (see Figure S3 and deposited CIF file).

Synthesis of [2][BF$_4$]

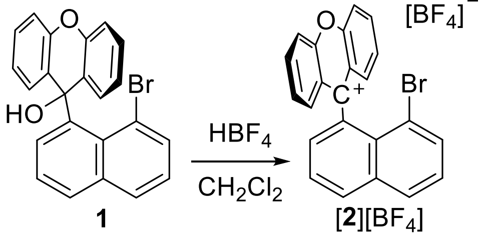

HBF$_4$•Et$_2$O (52% w/w in Et$_2$O, 243 mg, 1.44 mmol) was added to a solution of **1** (525 mg, 1.30 mmol) in CH_2Cl_2 (10 mL) at ambient temperature and exposed to air. The resulting mixture was stirred for 15 minutes during which the solution turned deep red. A red solid was subsequently obtained upon trituration with Et$_2$O (510 mg, 1.08 mmol, 83%). Single crystals suitable for X-ray crystal structure analysis were grown by layering hexane in a CH_2Cl_2 solution of [**2**][BF$_4$] under an ambient atmosphere. **^{1}H NMR** (499.41 MHz, CD_3CN) δ 8.58-8.55 (m, 2H, xanthylium-H), 8.44 (dd, J = 8.2, 1.0 Hz, 1H, naphthalene-H), 8.41 (d, J = 8.7 Hz, 2H, xanthylium-H), 8.30 (dd, J = 8.3, 0.8 Hz, 1H, naphthalene-H), 7.89-7.83 (m, 6H, xanthylium-H & naphthalene-H), 7.62 (t, J = 7.7 Hz, 1H, naphthalene-H), 7.57 (dd, J = 7.2, 1.1 Hz, 1H, naphthalene-H). **^{13}C{^{1}H} NMR** (125.76 MHz, CD_3CN) δ 179.07 (s, xanthylium-C), 158.70 (s, xanthylium-C), 145.41 (s, xanthylium-CH), 137.06 (s, naphthalene-C), 135.16 (s, naphthalene-CH), 134.53 (s, naphthalene-CH), 133.33 (s, naphthalene-CH), 132.46 (s, xanthylium-CH), 131.27 (s, naphthalene-C), 130.90 (s, naphthalene-CH), 130.45 (s, xanthylium-CH), 129.39 (s, naphthalene-CH), 129.17 (s, naphthalene-C), 126.67 (s, xanthylium-C), 126.56 (s, naphthalene-CH), 120.74 (s, xanthylium-CH), 119.70 (s, naphthalene-C). The identity of this compound was established by single-crystal X-ray diffraction (see Figure S6 and deposited CIF file).

Synthesis of 3

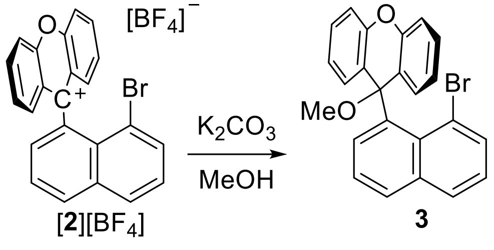

Potassium carbonate was added (844 mg, 6.11 mmol) at ambient temperature to a solution of [**2**][BF$_4$] (510 mg, 1.08 mmol) in MeOH (10 mL). The resulting mixture was stirred on the benchtop for 15 minutes, during which the solution turned pale-green. The solvent was removed under vacuum, and the crude product was washed with water and extracted with CH_2Cl_2 (3 × 30 mL). The organic fractions were combined, dried over

$MgSO_4$, and dried *in vacuo* to give **3** as a pale-green solid (430 mg, 1.03 mmol, 95%). Single crystals suitable for X-ray crystal structure analysis were grown by layering hexane in a CH_2Cl_2 solution of **3** under an ambient atmosphere. **^{1}H NMR** (499.41 MHz, CD_3CN) δ 8.81 (d, *J* = 7.2 Hz, 1H, naphthalene-*H*), 7.93 (dd, *J* = 8.0, 0.8 Hz, 1H, naphthalene-*H*), 7.84 (dd, *J* = 8.0, 0.8 Hz, 1H, naphthalene-*H*), 7.69 (t, *J* = 7.8 Hz, 1H, naphthalene-*H*), 7.50 (dd, *J* = 7.3, 0.9 Hz, 1H, naphthalene-*H*), 7.34 (td, *J* = 6.9, 1.6 Hz, 2H, methoxy-xanthene-*H*), 7.22 (dd, *J* = 8.1, 0.5 Hz, 2H, methoxy-xanthene-*H*), 7.11 (t, *J* = 7.8 Hz, 1H, naphthalene-*H*), 6.91 (td, *J* = 7.0, 1.0 Hz, 2H, methoxy-xanthene-*C*), 6.84 (dd, *J* = 7.8, 1.3 Hz, 2H, methoxy-xanthene-*C*) 2.70 (s, 3H, methoxy-xanthene-*H*). **^{13}C{^{1}H}NMR** (125.76 MHz, CD_3CN) δ 151.97 (s, methoxy-xanthene-*C*), 137.58 (s, naphthalene-*C*), 136.98 (s, naphthalene-*C*), 134.70 (s, naphthalene-*C*H), 131.35 (s, naphthalene-*C*), 131.16 (s, naphthalene-*C*H), 130.79 (s, naphthalene-*C*H), 129.68 (s, naphthalene-*C*H), 129.38 (s, methoxy-xanthene-*C*H), 128.63 (s, methoxy-xanthene-*C*H), 125.48 (s, naphthalene-*C*H), 125.15 (s, naphthalene-*C*H), 124.69 (s, methoxy-xanthene-*C*), 122.70 (s, methoxy-xanthene-*C*H), 118.92 (s, naphthalene-*C*), 115.81 (s, methoxy-xanthene-*C*H), 77.34 (s, methoxy-xanthene-*C*), 49.89 (s, methoxy-xanthene-*C*H_3). The identity of this compound was established by single crystal X-ray diffraction (see Figure S9 and deposited CIF file).

Synthesis of 4

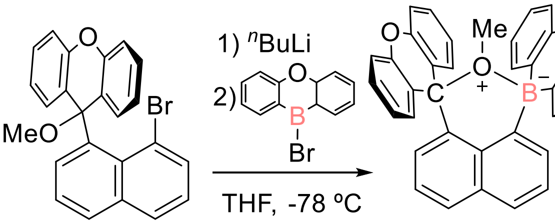

3 4

^{n}BuLi (0.40 mL, 1.00 mmol, 2.5 M in hexanes) was added to a solution of **3** (381 mg, 0.91 mmol) in THF (20 mL) at -78 °C leading to a color change from pale to olive green. The resulting solution was stirred at that temperature for 3 hours, at which point 10-bromo-9-oxa-10-boraanthracene (310 mg, 1.19 mmol) was added as a solid. The bright yellow mixture was warmed to room temperature and stirred overnight. The mixture was then quenched by adding DI water (10 mL). After removing the THF via rotary evaporation, the remaining aqueous phase was extracted with CH_2Cl_2 (3 × 30 mL). The organic fractions were combined, dried over $MgSO_4$, and evacuated to dryness to give a pale-yellow solid which was further purified by rinsing with acetonitrile. This procedure afforded **4** as a white solid (188 mg, 0.36 mmol, 40%). Single crystals suitable for X-ray crystal structure analysis were grown by layering pentane in a CH_2Cl_2 solution of **4** under an ambient atmosphere (see main text). **^{1}H NMR** (499.41 MHz, $CDCl_3$) δ 7.84 (t, *J* = 7.4 Hz, 2H, naphthalene-*H*), 7.50 (t, *J* = 8.1 Hz, 1H, naphthalene-*H*), 7.39 (d, *J* = 8.0 Hz, 2H, methoxy-xanthene-oxa-boraanthracene-*H*), 7.33-7.25 (m, 8H, naphthalene-*H*, methoxy-xanthene-oxa-boraanthracene-*H*), 7.19-7.16 (m, *J* = 4H, methoxy-xanthene-oxa-boraanthracene-*H*), 6.99-6.95 (m, 4H, methoxy-xanthene-oxa-boraanthracene-*H*), 6.69 (d, *J* = 8.0 Hz, 1H, naphthalene-*H*), 2.47 (s, 3H, methoxy-xanthene-oxa-boraanthracene-*H*). **^{13}C{^{1}H} NMR** (125.76 MHz, $CDCl_3$) δ 158.97 (s, methoxy-xanthene-oxa-boraanthracene-*C*), 150.83 (s, methoxy-xanthene-oxa-boraanthracene-*C*), 145.01 (s, naphthalene-*C*), 141.51 (s, methoxy-xanthene-oxa-boraanthracene-*C*), 135.85 (s, methoxy-xanthene-oxa-boraanthracene-*C*H), 132.69 (s, naphthalene-*C*H), 132.61 (s, naphthalene-*C*), 131.72 (s, methoxy-xanthene-oxa-boraanthracene-*C*), 130.91 (s, methoxy-xanthene-oxa-boraanthracene-*C*H), 130.60 (s, methoxy-xanthene-oxa-boraanthracene-*C*H), 129.58 (s, naphthalene-*C*H), 129.22 (s, methoxy-xanthene-oxa-boraanthracene-*C*H), 128.73 (s, naphthalene-*C*H), 128.32 (s, methoxy-xanthene-oxa-boraanthracene-*C*), 126.35 (s, naphthalene-*C*H), 126.08 (s, naphthalene-*C*H), 124.40 (s, naphthalene-*C*H), 124.14 (s, methoxy-xanthene-oxa-boraanthracene-*C*H), 122.18 (s, methoxy-xanthene-oxa-boraanthracene-*C*), 122.10 (s, methoxy-xanthene-oxa-boraanthracene-*C*H), 116.52 (s, methoxy-xanthene-oxa-boraanthracene-*C*H), 116.22 (s, methoxy-xanthene-oxa-boraanthracene-*C*H), 88.05 (s, methoxy-xanthene-oxa-boraanthracene-*C*), 53.66 (s, methoxy-xanthene-oxa-boraanthracene-*C*H_3). **^{11}B{^{1}H} NMR** (128.36 MHz, $CDCl_3$) δ 14.03 (methoxy-xanthene-oxa-boraanthracene-*B*). ESI-MS calcd for $^{12}C_{35}{}^{1}H_{22}{}^{11}B^{16}O_2^+$ [M]$^+$ 485.1707, found: 485.1704.

Synthesis of [5][BF4]

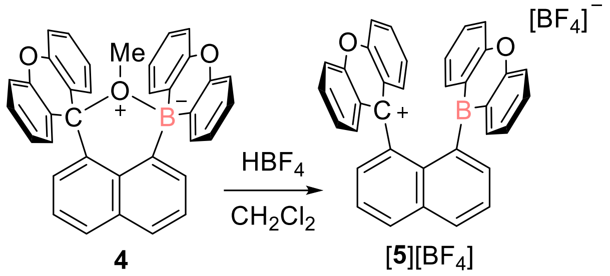

HBF_4•Et_2O (52% w/w in Et_2O, 36 mg, 0.21 mmol) was added to a solution of **4** (99 mg, 0.19 mmol) in CH_2Cl_2 (10 mL) at ambient temperature. The resulting mixture was stirred for 15 minutes on the bench top during which the solution turned dark red. A maroon solid was subsequently obtained upon trituration with Et_2O (100 mg, 0.17 mmol, 92%). Single crystals suitable for X-ray crystal structure analysis were grown by layering hexane in a CH_2Cl_2 solution of [**5**][BF_4] under an ambient atmosphere (see main text). **^{1}H NMR** (499.41 MHz, CD_2Cl_2) δ 8.52 (d, *J* = 8.1 Hz, 1H, naphthalene-*H*), 8.30 (d, *J* = 8.1 Hz, 1H, naphthalene-*H*), 8.06 (td, *J* = 7.2, 0.9 Hz, 2H, xanthylium-*H* & oxa-boraanthracene-*H*), 7.94 (t, *J* = 7.7 Hz, 1H, naphthalene-*H*), 7.83-7.81 (m, 3H, naphthalene-*H* & xanthylium-*H* & oxa-boraanthracene-*H*), 7.70-7.65 (m, 3H, naphthalene-*H* & xanthylium-*H* & oxa-boraanthracene-*H*), 7.61 (d, *J* = 6.5 Hz, 1H, naphthalene-*H*), 7.48-7.43 (m, 4H, xanthylium-*H* & oxa-boraanthracene-*H*), 7.26 (d, *J* = 8.4 Hz, 2H, xanthylium-*H* & oxa-boraanthracene-*H*), 7.02 (dd, *J* = 7.5, 1.0 Hz, 2H, xanthylium-*H* & oxa-boraanthracene-*H*), 6.78 (t, *J* = 7.21 Hz, 2H, xanthylium-*H* & oxa-boraanthracene-*H*). **^{13}C{^{1}H} NMR** (125.76 MHz, CD_2Cl_2) δ 176.44 (s, xanthylium-*C*), 159.03 (s, oxa-boraanthracene-*C*), 157.62 (s, xanthylium-*C*), 144.78 (s, xanthylium-*C*H), 138.85 (s, naphthalene-*C*) 135.95 (s, oxa-boraanthracene-*C*H), 135.86 (s, naphthalene-*C*), 135.56 (s, xanthylium-*C*H), 135.22 (s, naphthalene-*C*H), 134.91 (s, naphthalene-*C*), 133.75 (s, naphthalene-*C*H), 132.58 (s, xanthylium-*C*H), 131.57 (s, naphthalene-*C*H), 130.32 (s, naphthalene-*C*H), 130.32 (s, naphthalene-*C*), 129.96 (s, oxa-boraanthracene-*C*H), 127.12 (s, naphthalene-*C*H), 125.65 (s, naphthalene-*C*H), 124.77 (s, xanthylium-*C*), 123.33 (s, oxa-boraanthracene-*C*H), 119.68 (s, xanthylium-*C*H), 117.78 (s, oxa-boraanthracene-*C*H), oxa-boraanthracene-*C*-B was not detected. **^{11}B{^{1}H} NMR** (128.36 MHz, CD_2Cl_2) δ 51.92 (oxa-boraanthracene-*B*), -0.92 (*B*F_4^-). ESI-MS calcd for $^{12}C_{35}{}^1H_{22}{}^{11}B^{16}O_2^+$ $[M]^+$ 485.1707, found: 485.1708. Elemental analysis for $C_{35}H_{22}B_2F_4O_2$ (collected on the recrystallized sample) calculated: C: 73.47, H: 3.88; Found: C: 73.17, H: 3.92.

Synthesis of 5•

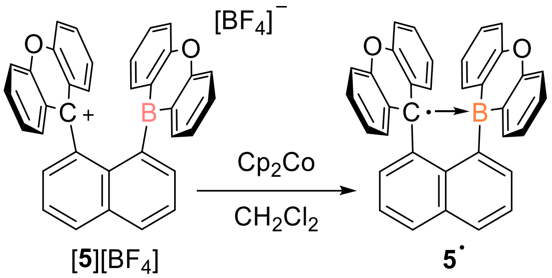

In a glovebox, cobaltocene (Cp_2Co) (34 mg, 0.18 mmol) was added into a solution of [**5**][BF_4] (103 mg, 0.18 mmol) in dry CH_2Cl_2 (5 mL). The resulting solution was stirred at ambient temperature for 1 hour, during which time the solution gradually turned dark red. The mixture was then triturated with Et_2O to afford **5•** as a red powder (86 mg). Single crystals suitable for X-ray analysis were grown by slow diffusion (hexanes/solution of **5•** in CH_2Cl_2) under a nitrogen atmosphere. Elemental analysis for $C_{35}H_{22}BO_2$ could not be obtained due to the reactivity of the radical in the presence of oxygen. The identity of this compound was established by single-crystal X-ray diffraction (see main text).

Synthesis of [K(18-c-6)][5]

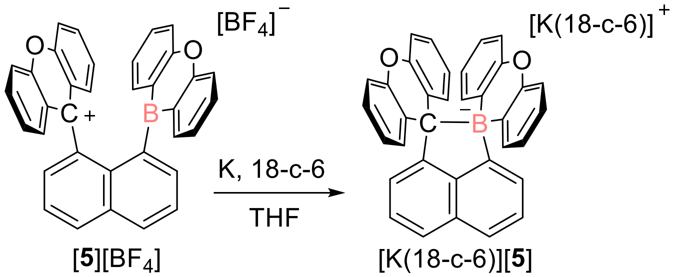

In a glovebox, [**5**][BF_4] (46.9 mg, 0.08 mmol) and 18-crown-6 (18-c-6) (21.6 mg, 0.08 mmol) were dissolved in dry THF (5.0 mL). To this solution, a small piece of potassium was added, and the mixture was stirred at room temperature for 20 min, at which point the potassium was removed. The dark turquoise solution was then triturated with Et_2O to afford [K(18-c-6)][**5**] as a pale beige powder (38.8 mg, 0.05 mmol, 61%). Single crystals suitable for X-ray analysis were grown by slow diffusion (hexanes/solution of [K(18-c-6)][**5**] in CH_2Cl_2) under a nitrogen atmosphere (see main text). **^{1}H NMR** (499.41 MHz, CD_2Cl_2) δ 7.73 (d, *J* = 8.1 Hz, 1H, naphthalene-*H*), 7.66 (d, *J* = 8.1 Hz, 1H, naphthalene-*H*), 7.51-7.46 (m, 2H, naphthalene-*H*), 7.13 (d, *J* = 6.4 Hz, 1H, naphthalene-*H*), 7.07 (d, *J* = 6.8 Hz, 1H, naphthalene-*H*), 6.71 (td, *J* = 6.7, 1.5 Hz, 2H, xanthene-*H*), 6.58-6.55 (m, 4H, xanthene-*H* & oxa-borateanthracene-*H*), 6.44 (d, *J* = 7.3 Hz, 2H, xanthene-*H*), 6.36-6.30 (m, 4H, xanthene-*H* & oxa-borateanthracene-*H*), 6.24 (d, *J* = 6.3 Hz, 2H, oxa-borateanthracene-*H*), 5.97 (dd, *J* = 7.7, 1.0 Hz, 2H, oxa-borateanthracene-*H*), 3.49 (s, 24H, 18-crown-6-*H*). **^{13}C{^{1}H} NMR** (125.76 MHz, CD_2Cl_2) δ 158.12 (s, xanthene-*C*), 155.88 (s, naphthalene-*C*), 151.92 (s, oxa-borateanthracene-*C*), 148.62 (s, naphthalene-*C*), 137.86 (s, xanthene-*C*), 135.14 (s, oxa-borateanthracene-*C*H), 131.75 (s, naphthalene-*C*), 128.45 (s, naphthalene-*C*H), 127.70 (s, naphthalene-*C*H), 127.53 (s, naphthalene-*C*H), 127.00 (s, oxa-borateanthracene-*C*H), 124.07 (s, xanthene-*C*H), 123.63 (s, naphthalene-*C*H), 123.12 (s, naphthalene-*C*H), 122.63 (s, xanthene-*C*H), 121.46 (s, naphthalene-*C*H), 121.30 (s, xanthene-*C*H), 120.40 (s, oxa-borateanthracene-*C*H), 113.90 (s, xanthene-*C*H), 113.84 (s, oxa-borateanthracene-*C*H), 70.60 (s, 18-crown-6-*C*H_2), oxa-borateanthracene-*C*-B, naphthalene-*C*-B and xanthene-*C*-B were not detected. **^{11}B{^{1}H} NMR** (128.36 MHz, CD_2Cl_2) δ -4.58 (oxa-borateanthracene-*B*). ESI-MS calcd for $^{12}C_{35}{}^{1}H_{22}{}^{11}B^{16}O_2^-$ $[M]^-$ 485.1718, found: 485.1724. Elemental analysis for $C_{47}H_{46}BO_8K{\bullet}(CH_2Cl_2)_{0.7}$ (collected on the recrystallized sample) calculated: C: 67.54, H: 5.63; Found: C: 67.74, H: 5.54.

NMR spectra and crystal structures

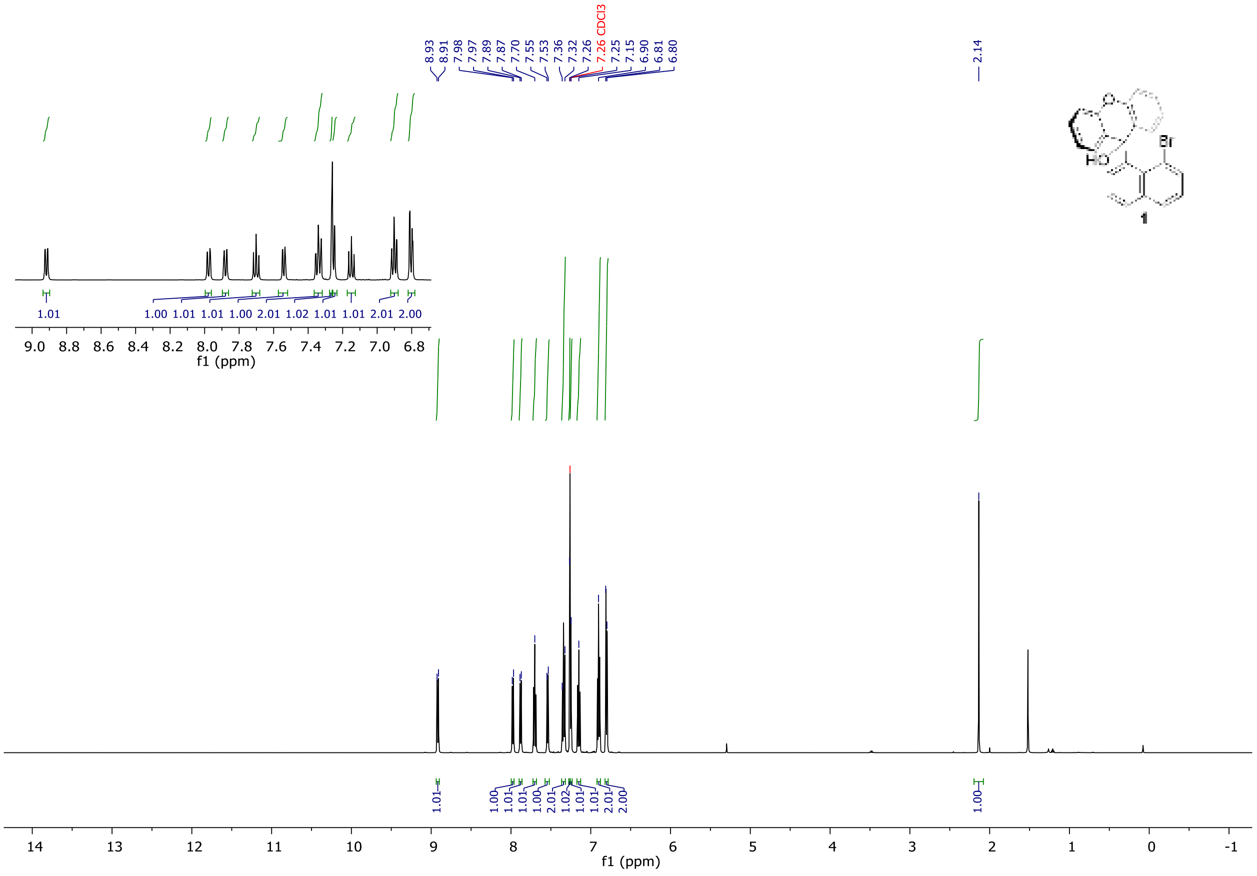

Figure S1. ^{1}H NMR spectrum of **1** in $CDCl_3$.

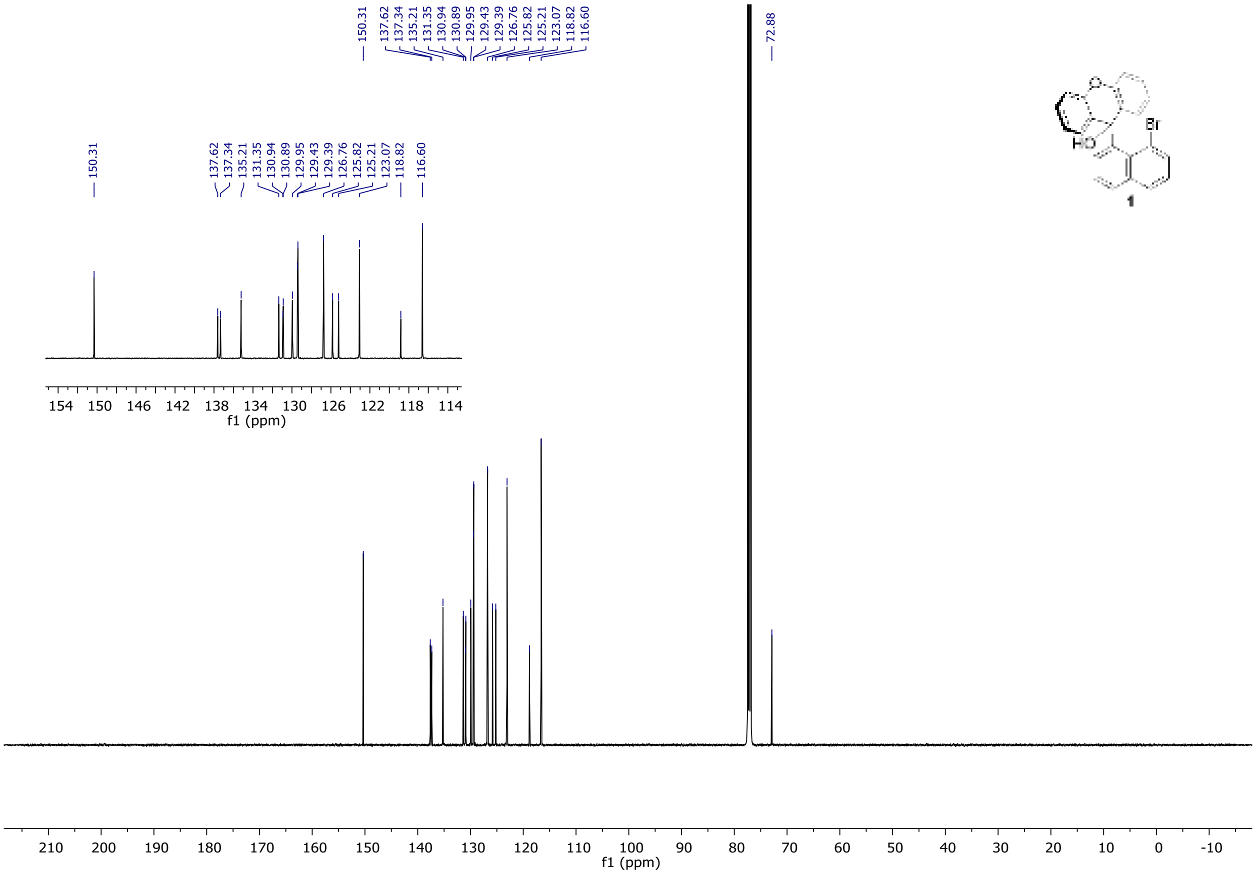

1

Figure S2. $^{13}C\{^{1}H\}$ NMR spectrum of **1** in $CDCl_3$. The solvent peak is truncated.

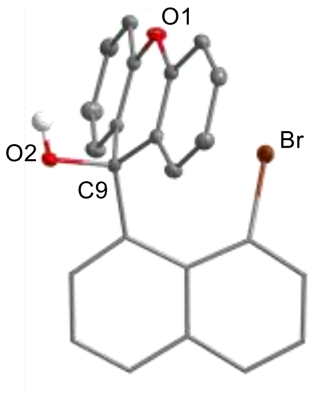

Figure S3. Solid state structure of **1**. Ellipsoids are drawn at the 50% probability level. Hydrogen atoms are omitted for clarity. (Gray: C, red: O, brown: Br, white: H)

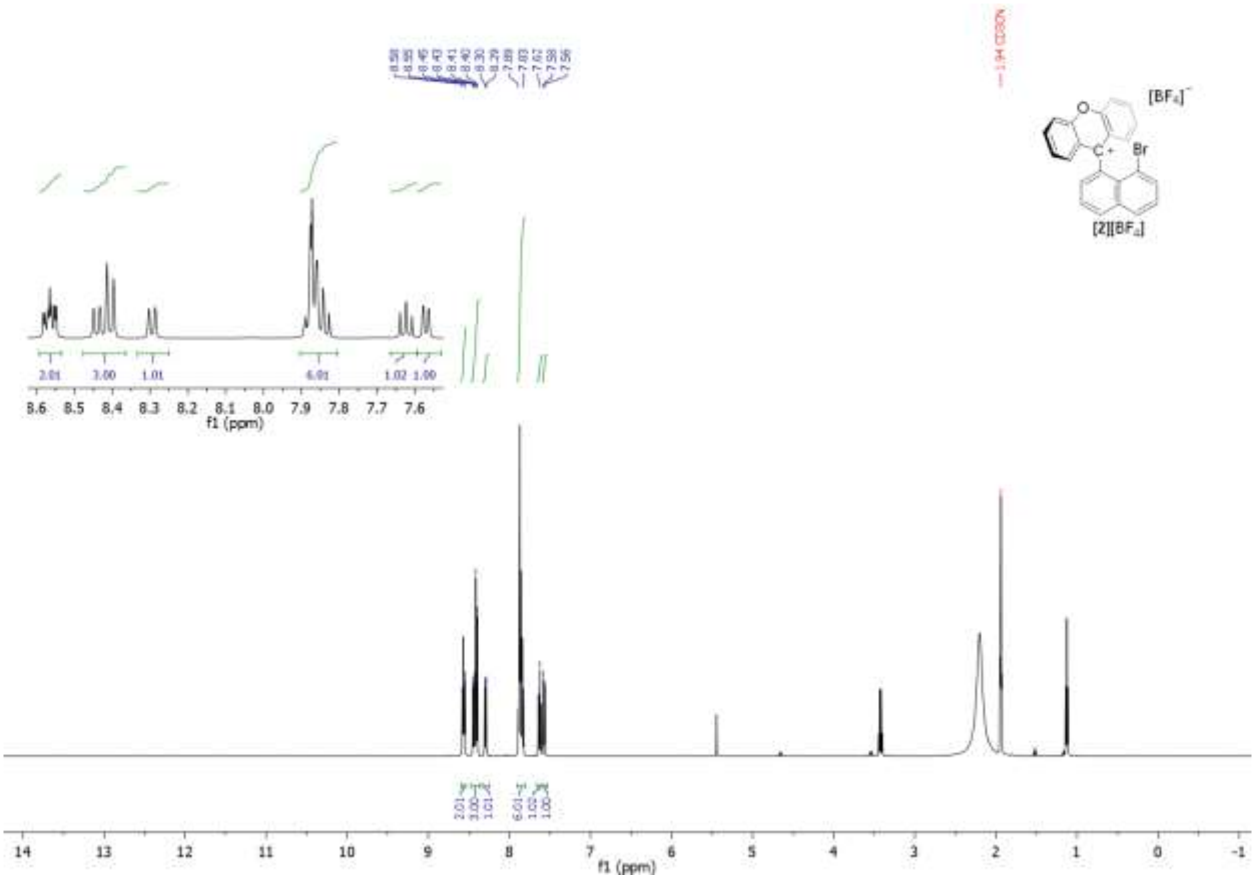

Figure S4. ^{1}H NMR spectrum of [**2**][BF_4] in CD_3CN.

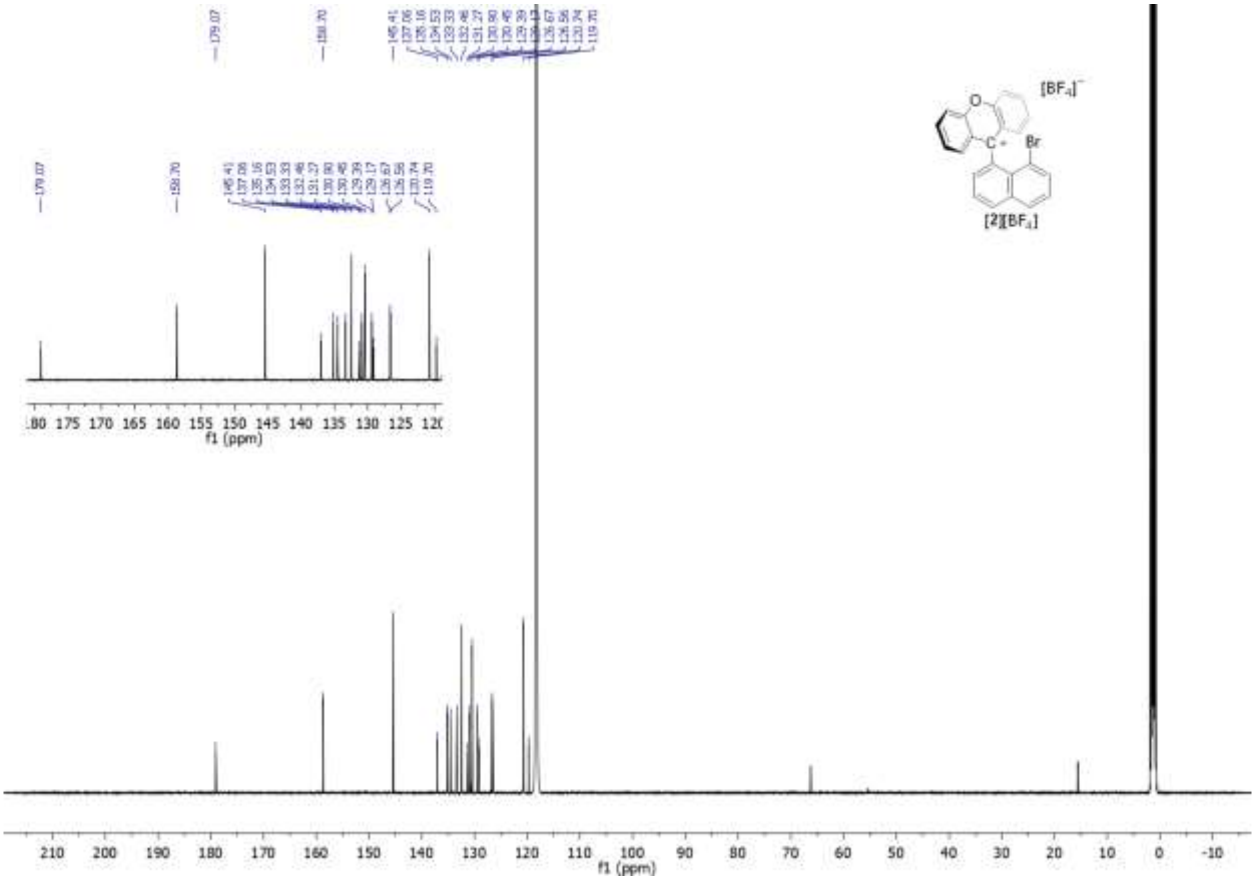

Figure S5. $^{13}C\{^{1}H\}$ NMR spectrum of [**2**][BF_4] in CD_3CN. The solvent peak is truncated.

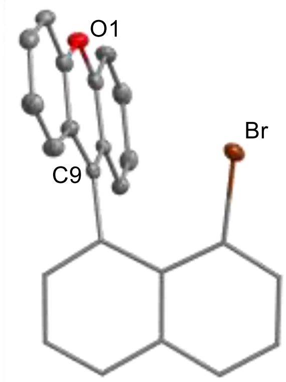

Figure S6. Solid state structure of [**2**][BF_4]. Ellipsoids are drawn at the 50% probability level. Counterion and hydrogen atoms are omitted for clarity. (Gray: C, red: O, brown: Br).

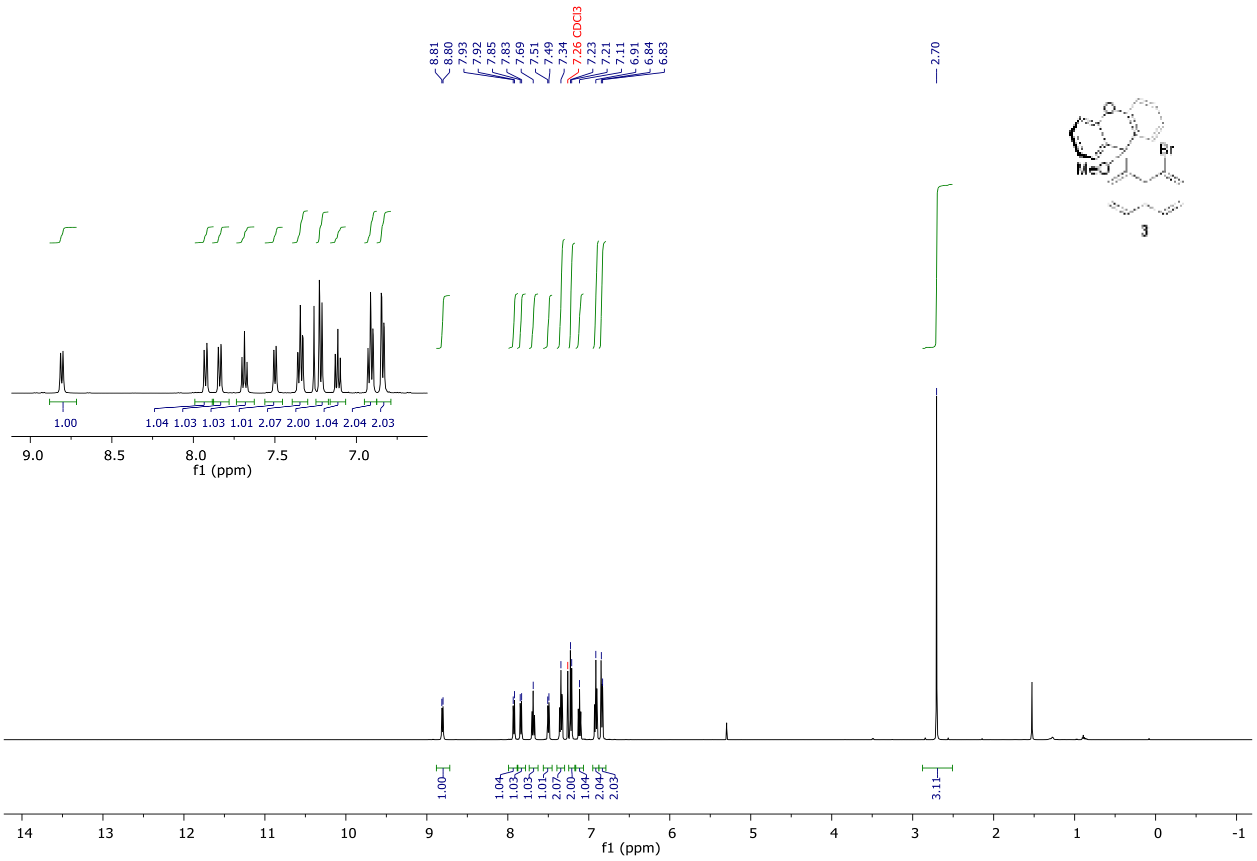

Figure S7. ^{1}H NMR spectrum of **3** in $CDCl_3$.

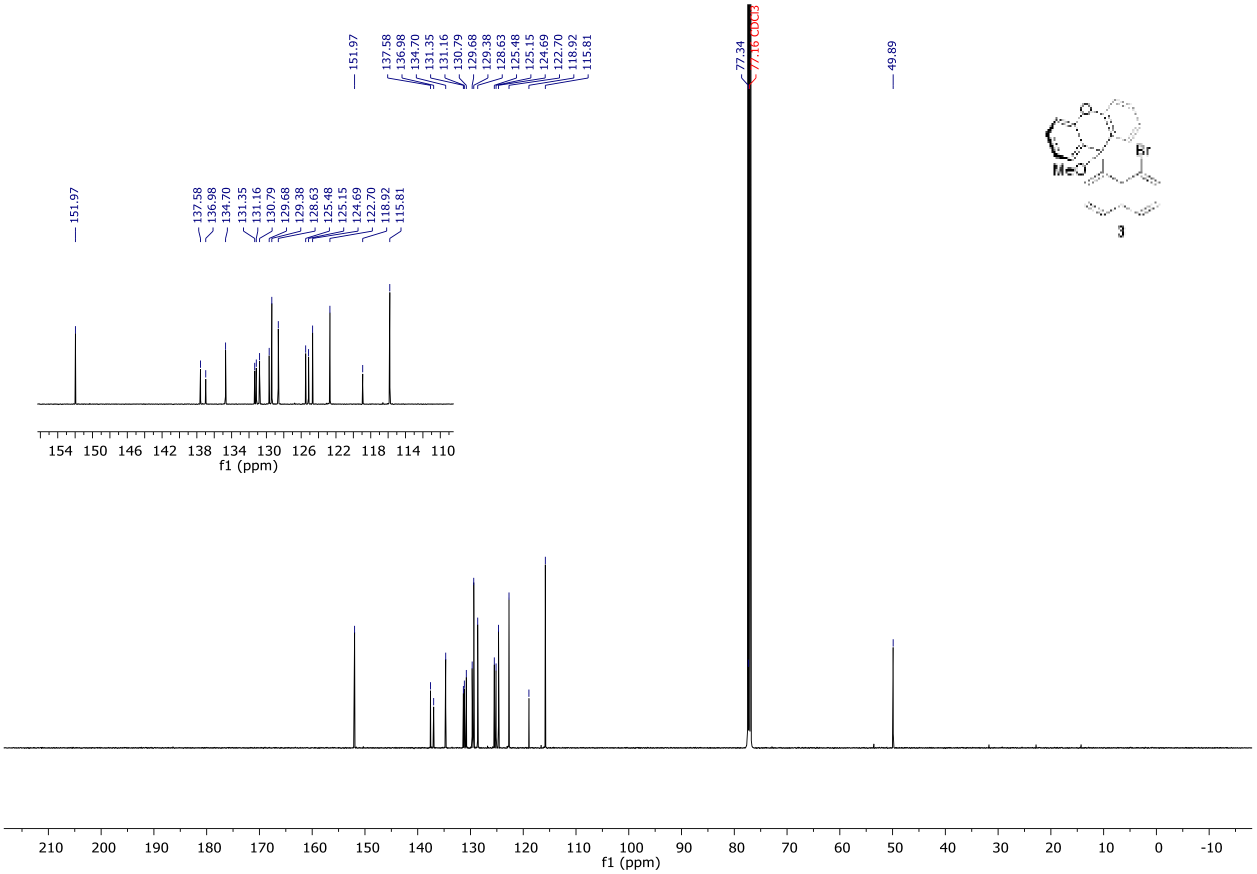

Figure S8. $^{13}C\{^{1}H\}$ NMR spectrum of **3** in $CDCl_3$. The solvent peak is truncated.

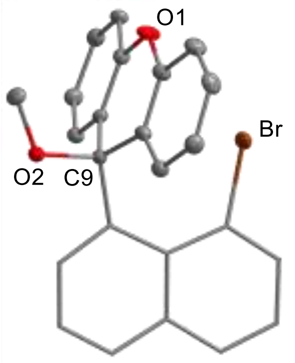

Figure S9. Solid state structure of **3**. Ellipsoids are drawn at the 50% probability level. Hydrogen atoms are omitted for clarity. (Gray: C, red: O, brown: Br).

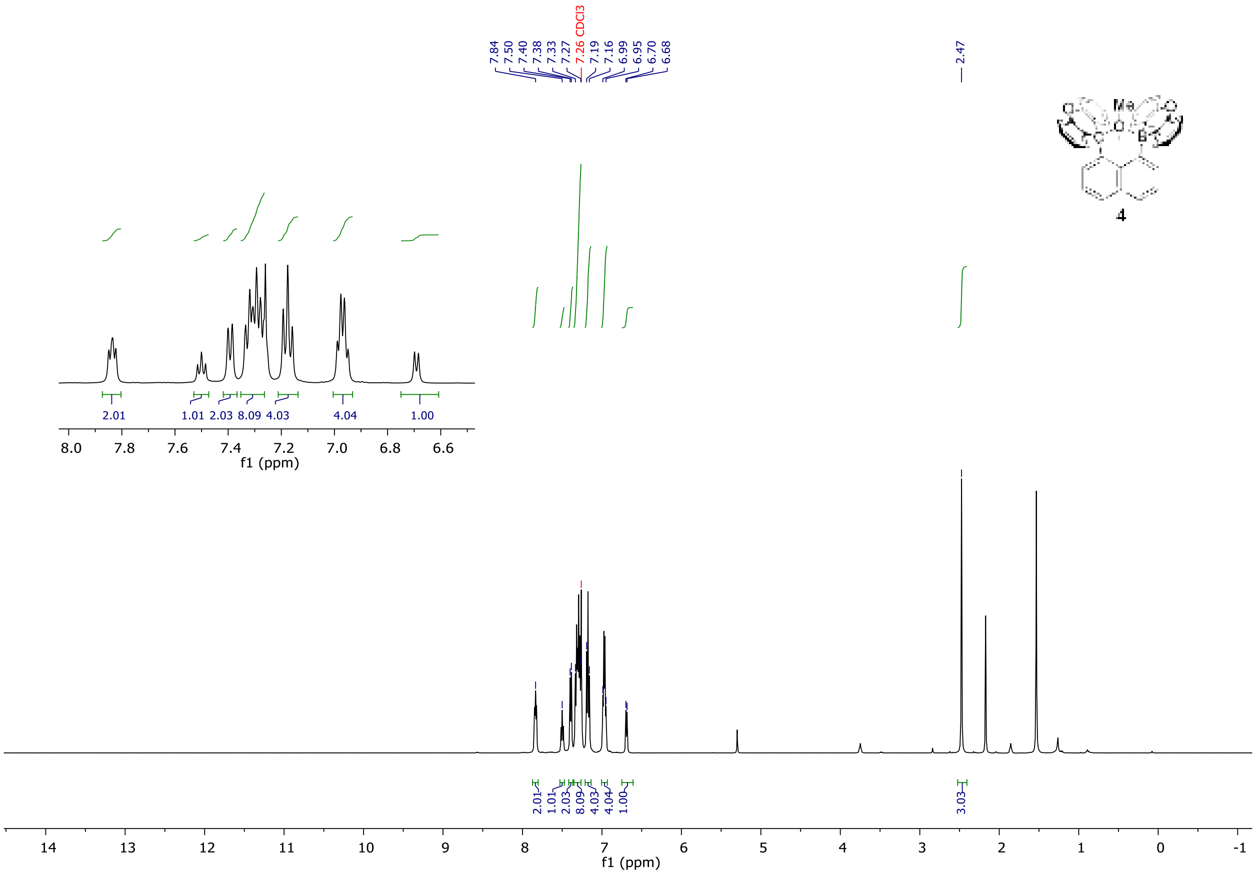

Figure S10. ^{1}H NMR spectrum of **4** in $CDCl_3$.

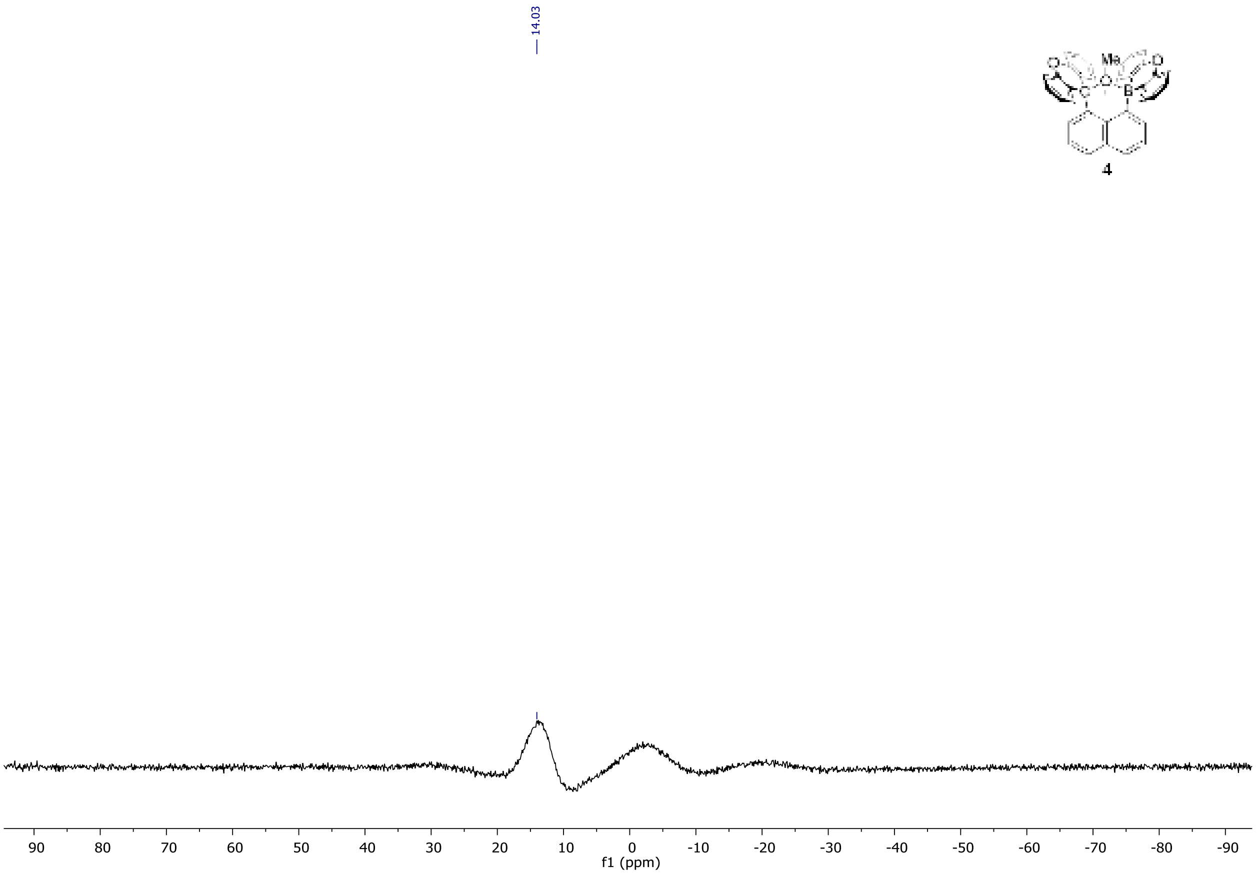

Figure S11. $^{11}B\{^{1}H\}$ NMR spectrum of **4** in $CDCl_3$.

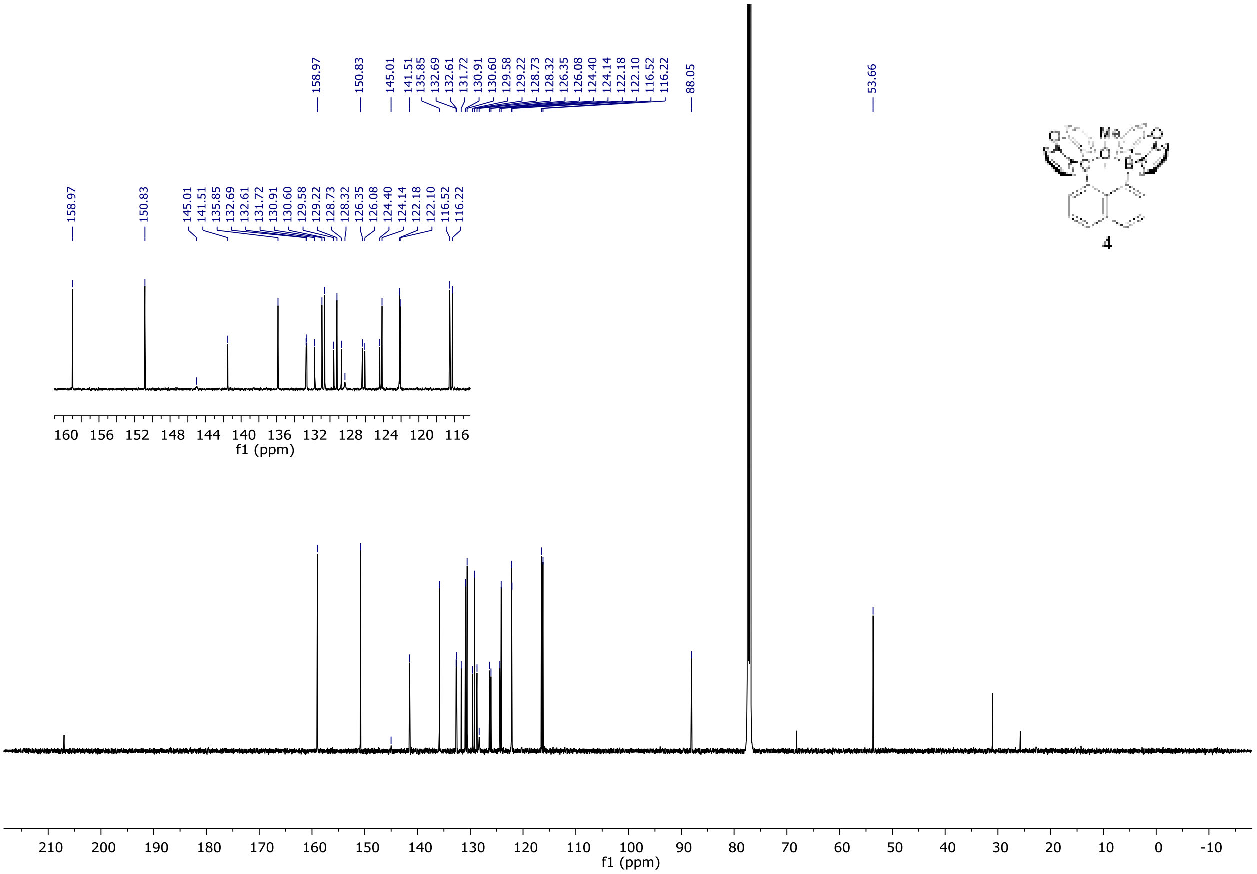

4

Figure S12. $^{13}C\{^1H\}$ NMR spectrum of **4** in $CDCl_3$. The solvent peak is truncated.

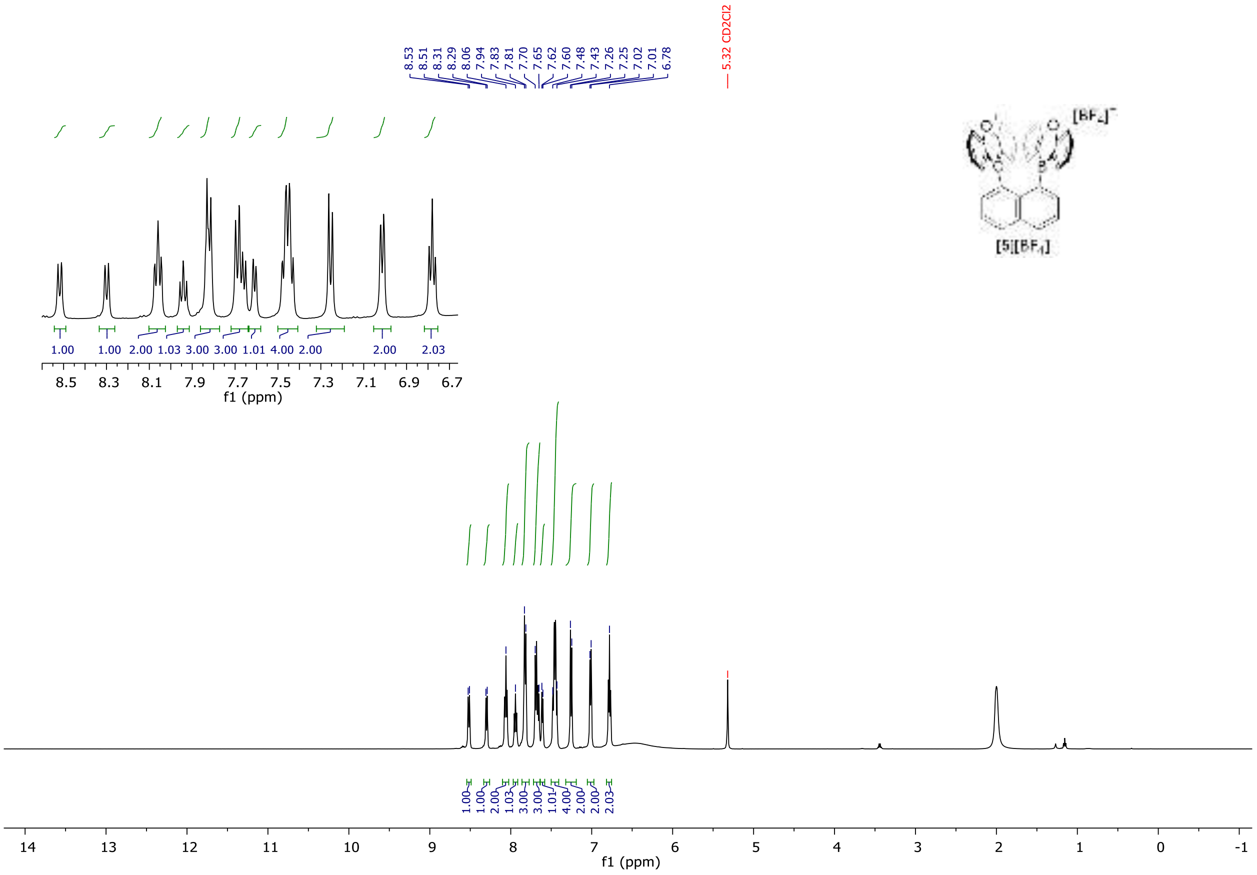

Figure S13. ^{1}H NMR spectrum of [**5**][BF_4] in CD_2Cl_2.

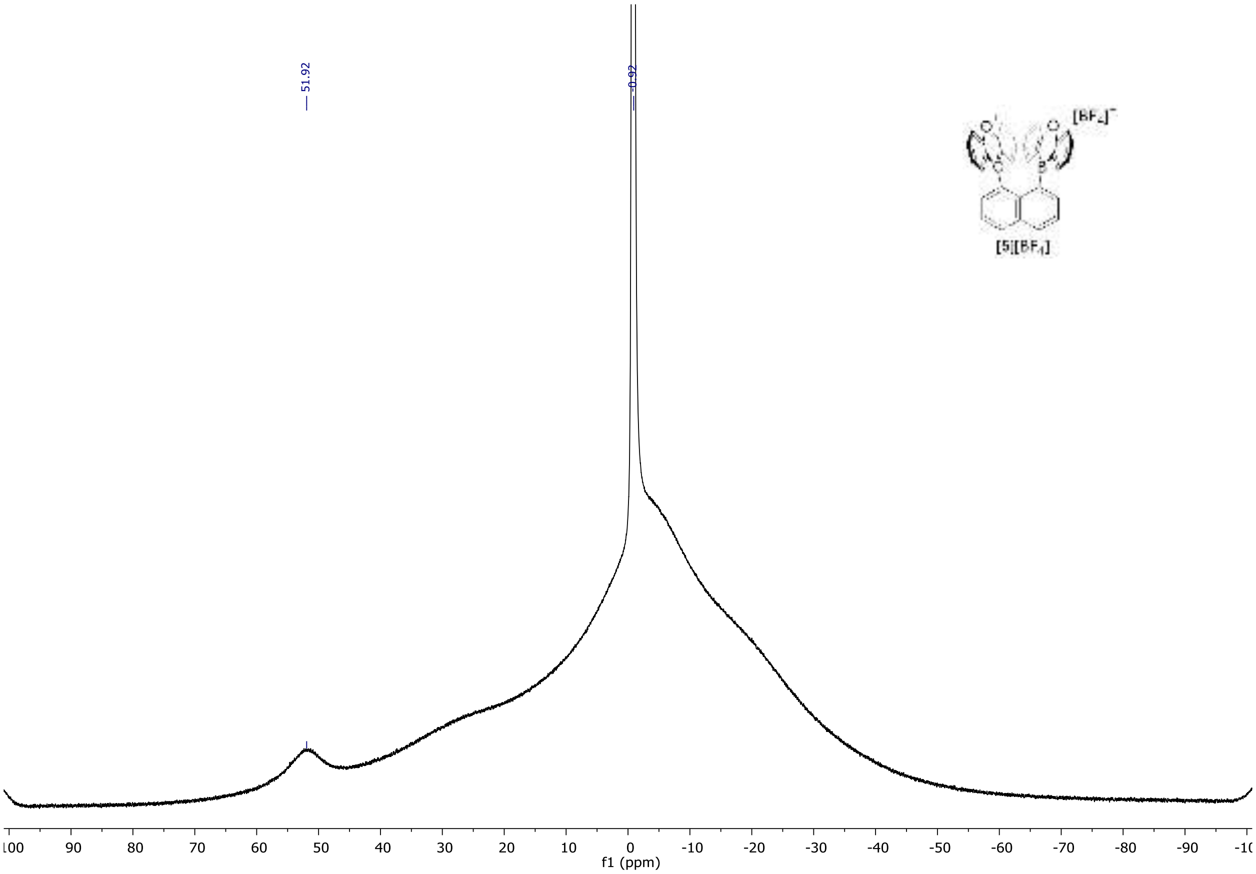

Figure S14. Uncorrected $^{11}B\{^{1}H\}$ NMR spectrum of [**5**][BF_4] in CD_2Cl_2.

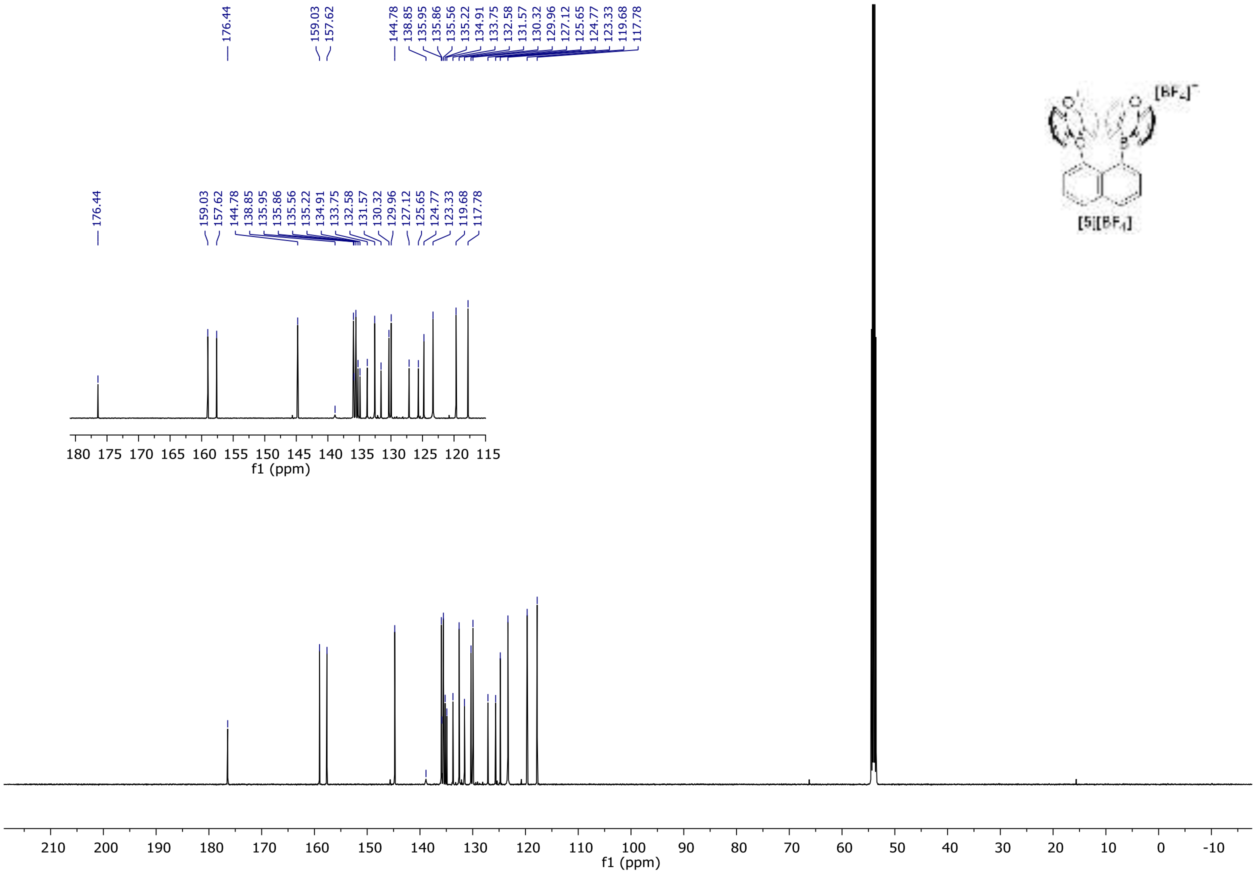

Figure S15. $^{13}C\{^1H\}$ NMR spectrum of [**5**][BF_4] in CD_2Cl_2. The solvent peak is truncated.

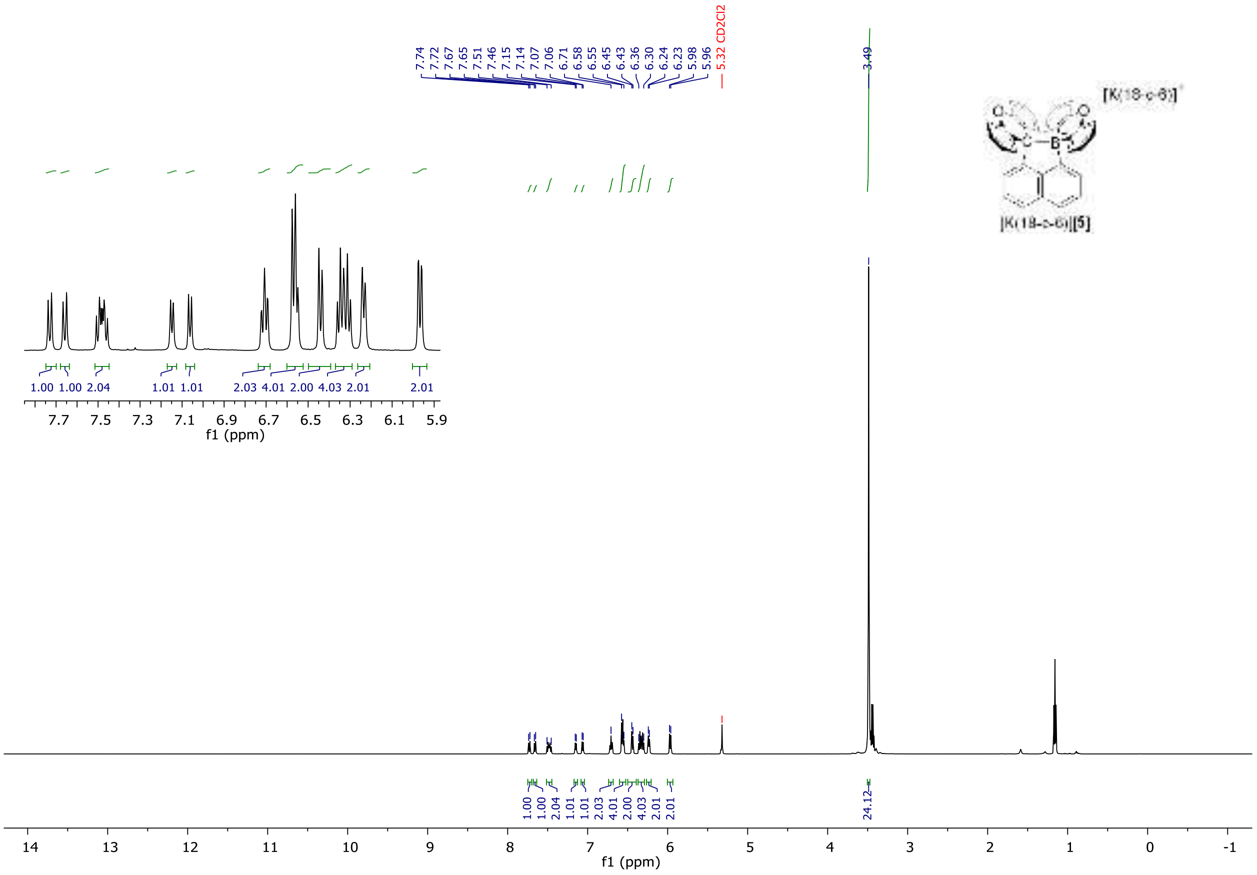

Figure S16. ^{1}H NMR spectrum of [K(18-c-6)][**5**] in CD_2Cl_2.

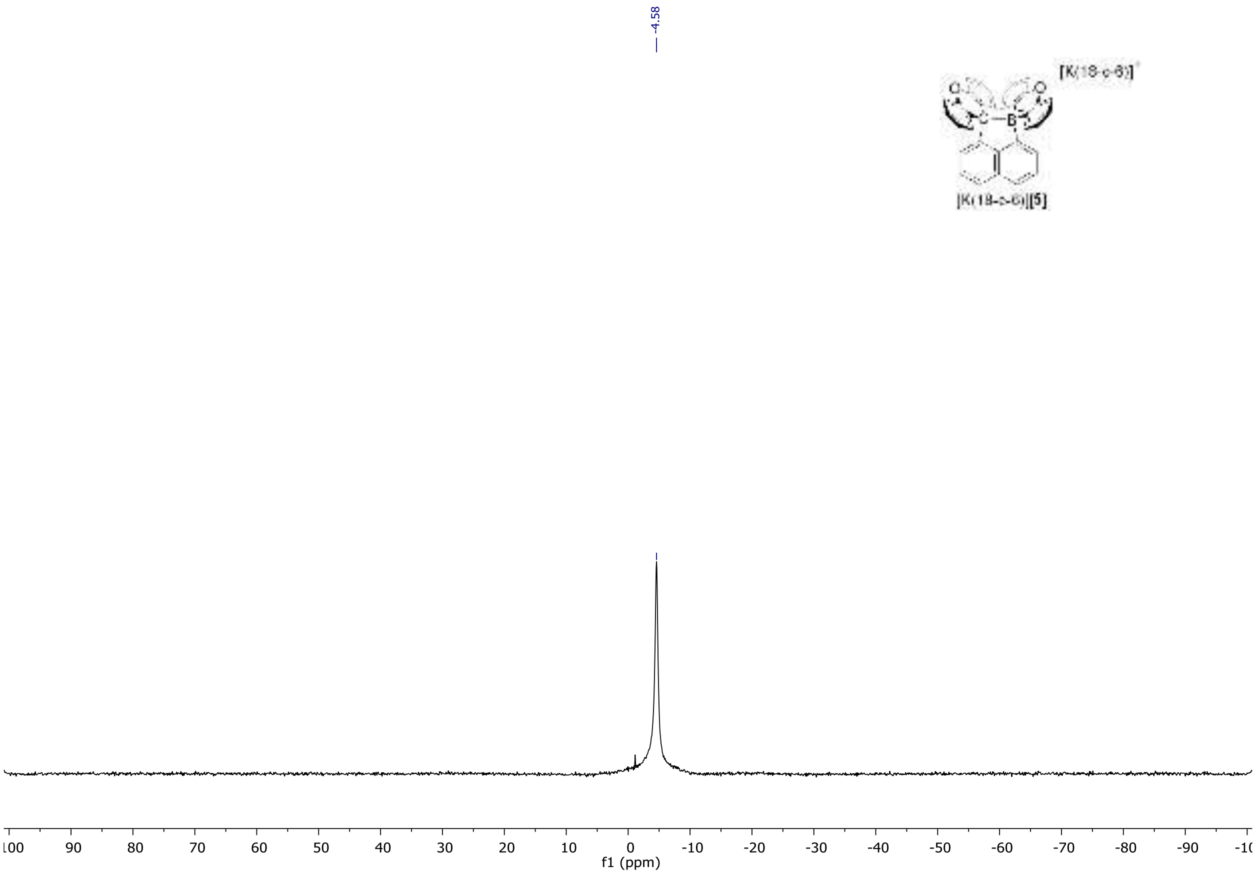

Figure S17. $^{11}B\{^{1}H\}$ NMR spectrum of [K(18-c-6)][**5**] in CD_2Cl_2.

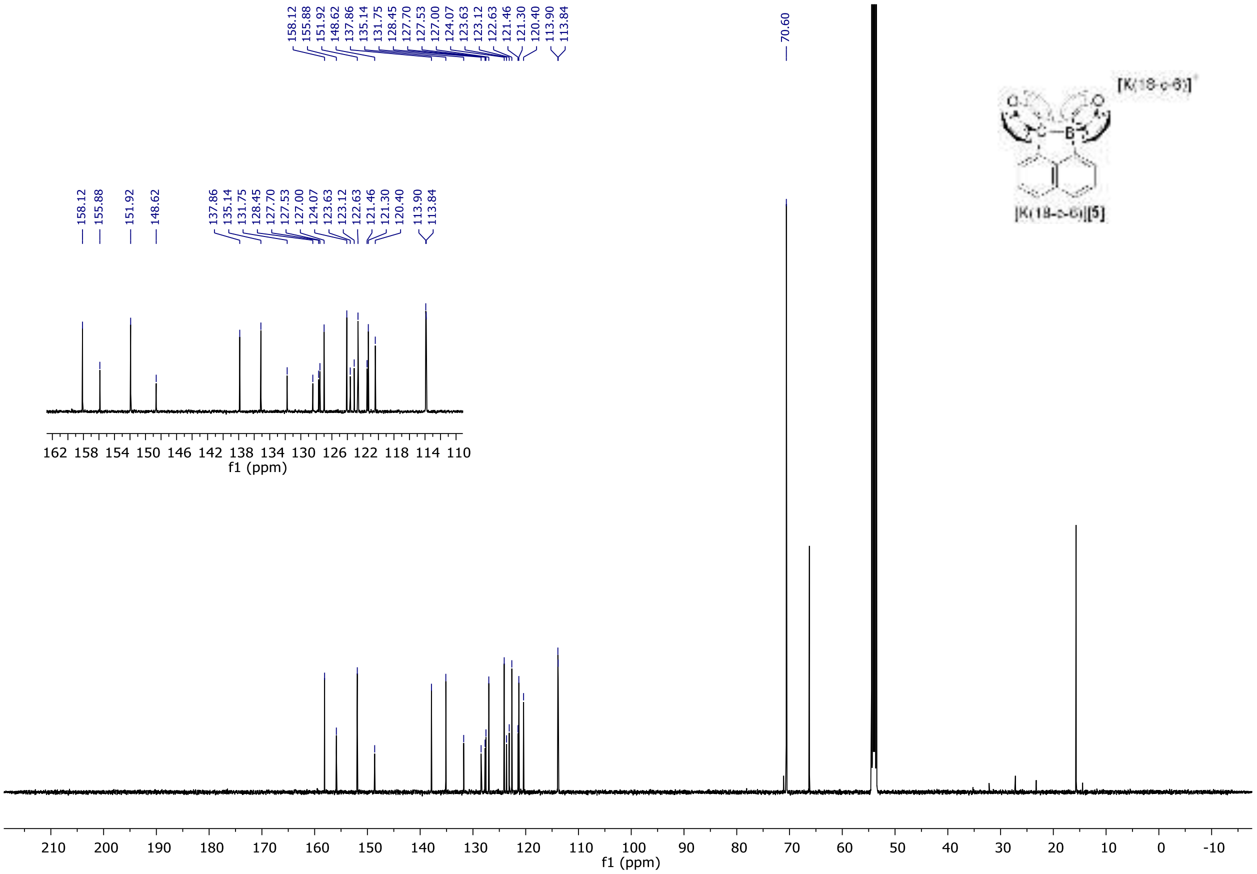

Figure S18. $^{13}C\{^1H\}$ NMR spectrum of [K(18-c-6)][**5**] in CD_2Cl_2.The solvent peak is truncated.

Cyclic Voltammetry and Differential Pulse Voltammetry

Cyclic Voltammetry (CV) measurements were recorded with a CH Instrument (Model 660 D) electrochemical analyzer, using a glassy carbon working electrode, a Ag/AgCl reference electrode, a platinum wire auxiliary electrode, and a 1 mM solution of [**5**][BF_4] in degassed dichloromethane with 0.25 M $TBAPF_6$ as the supporting electrolyte. The voltammogram was collected at a scan rate of 0.1 V/s. The potential was referenced against Fc/Fc^+ in the same solvent. All CV measurements were carried out at room temperature under N_2.

EPR spectroscopy

EPR measurements were carried out on a Bruker EMX X-band spectrometer. A sample containing a 10^{-4} M concentration of **5**$^{\bullet}$ was prepared using dichloromethane as a solvent inside a glove box. The resulting solution was then transferred to a 2 mm EPR tube. The EPR spectrum was obtained at room temperature with a microwave power of 1.987 mW, a modulation amplitude of 0.1 G, a time constant of 163.84 ms, and a sweep time of 60.0 seconds.

The EPR spectrum was simulated using the EasySpin[7] software package in MATLAB, yielding the hyperfine coupling constants listed in the following figure.

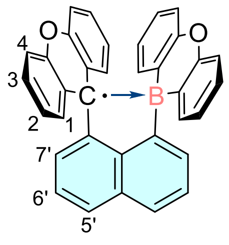

g = 2.0034

Hyperfine in Gauss

	Exp	Comp
H1	3.16	3.16
H2	0.54	0.94
H3	3.28	3.68
H4	0.51	0.92
H5'	0.31	0.53
H6'	0.36	0.76
H7'	0.39	0.77
^{11}B	0.65	0.67
^{10}B	0.22	

Figure S19. Hyperfine coupling constants and g value of **5**$^{\bullet}$ used for the simulated EPR spectrum and calculated values obtained through DTF calculations. The ^{10}B hyperfine coupling is calculated using the ratio of the gyromagnetic ratio of the two isotopes (4.6 MHz/T for ^{10}B and 13.7 MHz/T for ^{11}B).

UV-vis spectroscopy

Two stock solutions (0.03 mM) of [**5**][BF_4] (purple solution) and [K(18-c-6)][**5**] (pale yellow solution) were prepared in dry and degassed dichloromethane. Samples of 3 mL of each solution were transferred into a quartz cuvette equipped with a Teflon stopcock to record the UV-vis spectrum of these two individual species (Figures S20 and S21). A sample of **5**$^{\bullet}$ was prepared in a Jenway quartz cuvette (2 mm path length) by combining a 0.5 mL aliquot of a stock solution of [**5**][BF_4] (0.4 mM) with a 0.5 mL aliquot of a stock solution of [K(18-c-6)][**5**] (0.4 mM). The resulting solution was agitated for 10 min to generate a red solution of the radical, the spectrum of which was recorded (Figure S22).

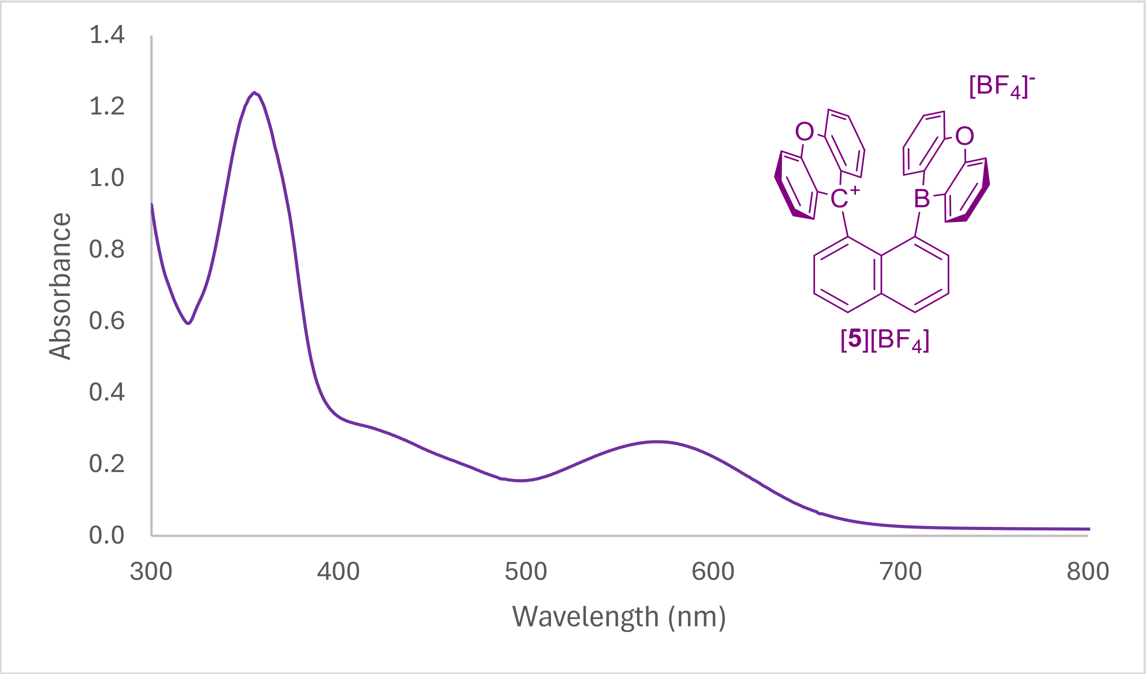

Figure S20. UV-vis spectrum of [**5**][BF_4].

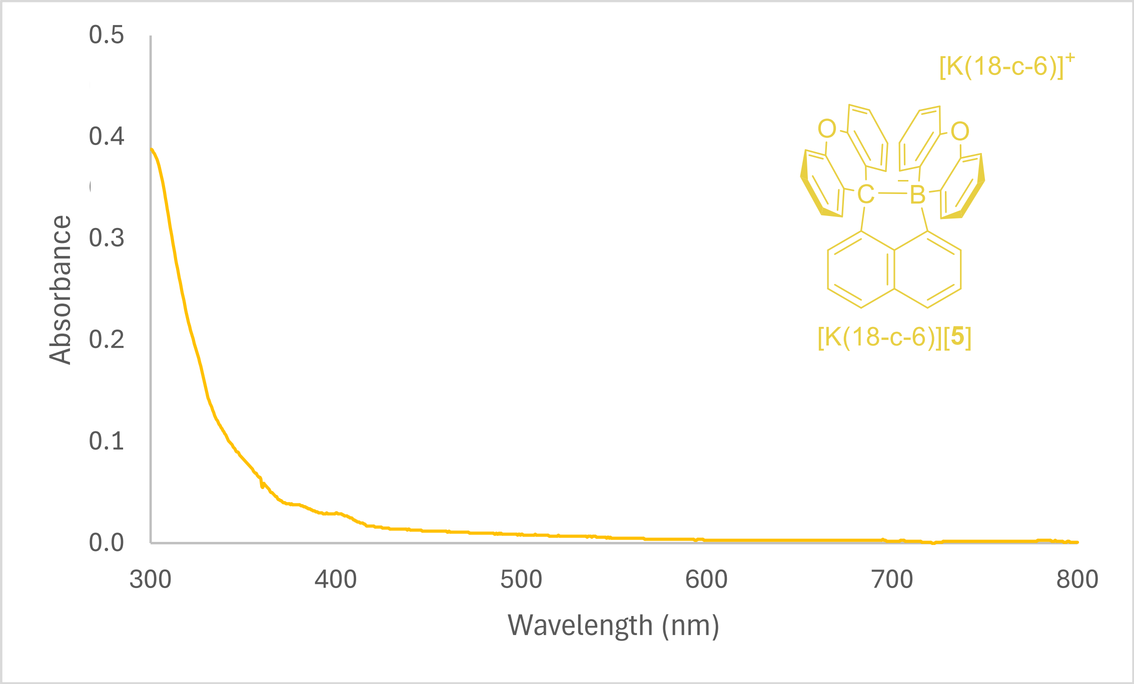

Figure S21. UV-vis spectrum of [K(18-c-6)][**5**].

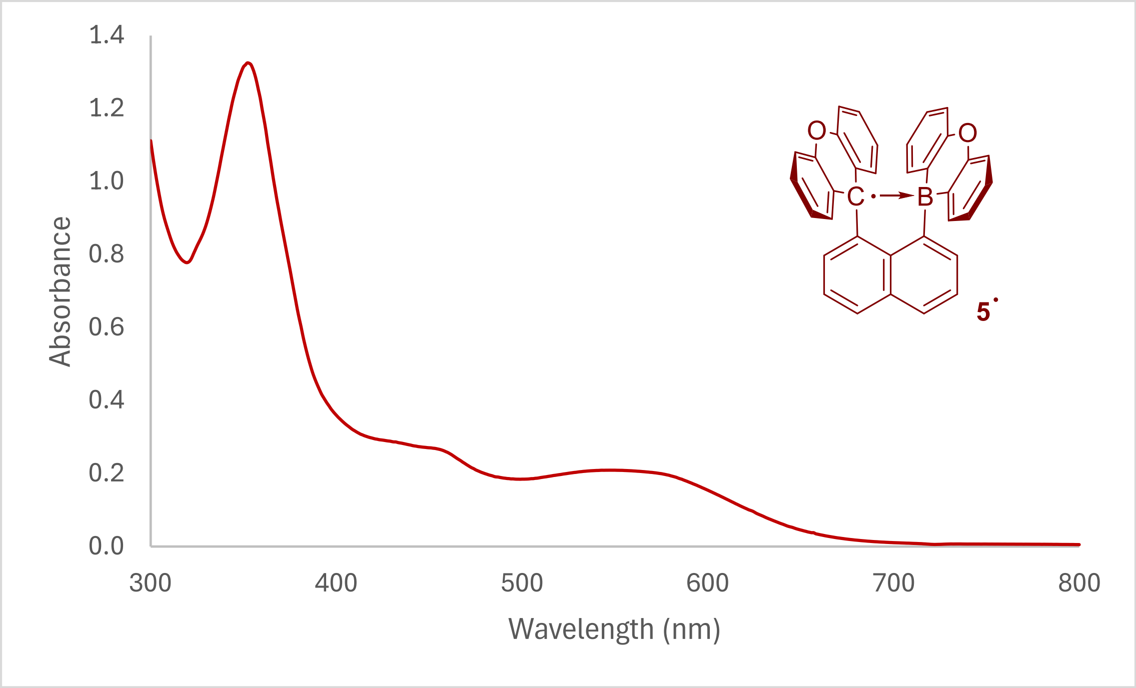

Figure S22. UV-vis spectrum of **5**•.

Spectroelectrochemical Analysis

Spectroelectrochemical measurements were carried out with a Pine instrument WaveNow potentiostat using a platinum honeycomb spectroelectrochemical electrode card, featuring a working and counter electrode. This electrode card was placed into a matching quartz cuvette which was then fitted with a Ag/AgCl gel reference electrode. A dichloromethane solution of [**5**][BF_4] (0.30 mM) containing $TBAPF_6$ (0.25 M) was prepared and used for the measurement which was conducted under N_2. A UV-vis spectrum was taken every 20 seconds, while the potential was scanned from positive potentials to negative potentials, at the rate of 1 mV/s, starting at 400 mV and ending at -600 mV. For enhanced visualization, the resulting spectra in Figure S23 have been color-coded.

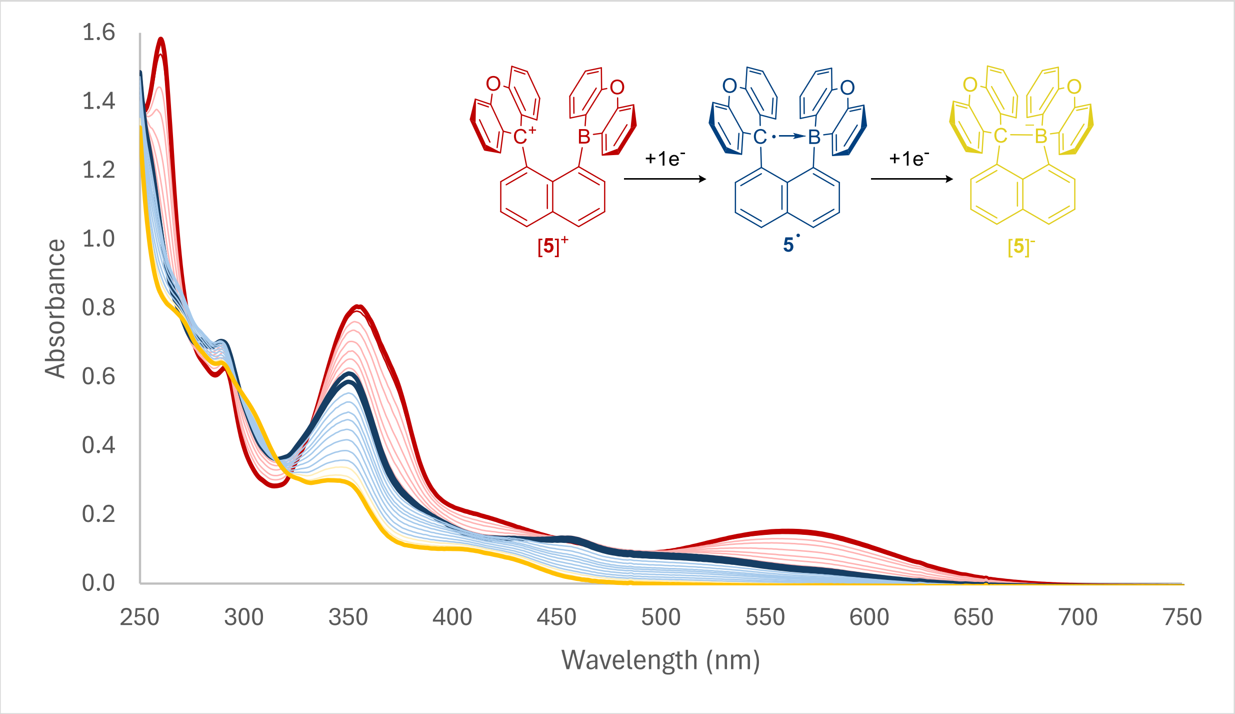

Figure S23. UV-vis-EC spectrum of [**5**][BF_4] in dichloromethane. Color coding: cation [**5**]$^+$ (red), neutral radical **5**$^{\bullet}$ (blue), anion [**5**]$^-$ (yellow).

Computational Analysis

Computations were implemented using Density Functional Theory (DFT) in the Gaussian 16 program.[8] All calculations were conducted with the B3LYP functional (uB3LYP for **5**$^{\bullet}$) and 6-311G(d,p) as the basis set. In all cases, the crystal structure geometry was chosen as the starting point for the optimization and the Polarizable Continuum Model (PCM) (solvent = dichloromethane) was applied. No imaginary frequencies were found for the optimized structures at the same level of theory with application of the PCM (solvent = dichloromethane), confirming that a local minimum on the potential energy hypersurface had, in all cases, been reached. The spin density of **5**$^{\bullet}$ was visualized using the GaussView 5.0 program. The optimized structures were used for the Time-Dependent Density Functional Theory (TD-DFT) calculations, the EPR calculations, and the NBO analysis (using NBO 7)[9] . The TD-DFT output of [**5**]$^+$, **5**$^{\bullet}$ and [**5**]$^-$ was plotted using GaussSum 3.0. The NBOs and Kohn-Sham orbitals were visualized and plotted using the Avogadro program.[10] QTAIM and Pipek-Mezey calculations were carried out on the wave functions derived from the optimized structures using the Multiwfn[11] program and visualized using VMD (Visual Molecular Dynamics)[12] and the Avogadro program, respectively.

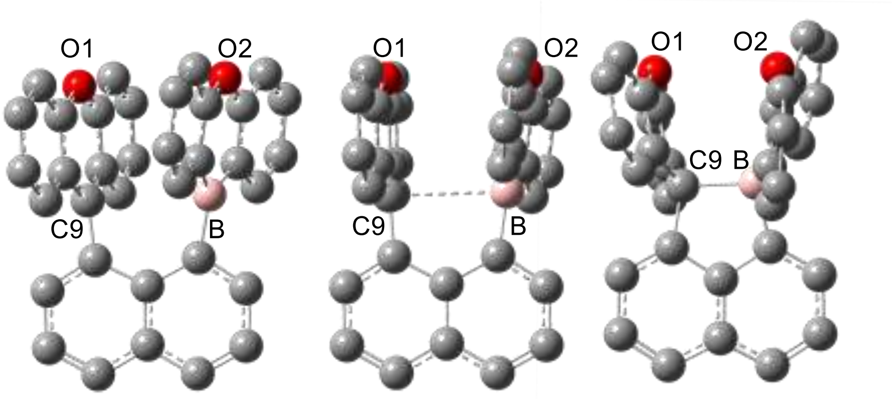

[**5**][BF_4] **5**$^{\bullet}$ [K(18-c-6)][**5**]

Figure S24. Geometry optimized structures of [**5**][BF_4], **5**$^{\bullet}$ and [K(18-c-6)][**5**].

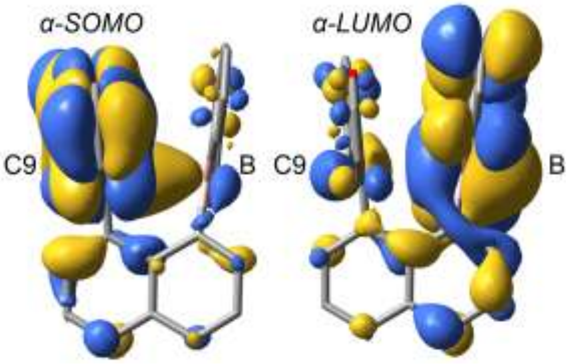

Figure S25. α-SOMO and α-LUMO orbitals for compound **5**$^{\bullet}$ (isosurface value of 0.02).

Table S1. XYZ coordinates of the optimized geometry of [**5**][BF_4].

Atom	Coordinates			Atom	Coordinates		
	X	Y	Z		X	Y	Z
O	-2.492567	1.507140	-1.135029	H	1.729514	0.163591	-2.250945
O	-0.318397	2.474169	1.482169	C	-2.794430	-2.078407	-0.366780
C	-0.135933	0.080541	1.676863	H	-2.196639	-2.980372	-0.320512
C	-0.859953	1.291420	1.833872	C	1.931896	-4.272898	-1.214515
C	-2.166651	-0.875508	-0.761581	H	2.563653	-5.150306	-1.289364
C	1.466409	-2.088498	-0.151521	C	1.294136	2.220696	-2.645064
C	1.899159	-1.116143	0.818116	H	2.273329	2.437387	-3.052473
C	2.273466	-3.272240	-0.270945	C	1.439789	3.833530	0.769667
C	-1.224764	1.681781	-1.610594	H	0.796045	4.687313	0.929975
C	0.953177	2.556309	1.042159	C	3.049330	1.569850	0.319798
C	0.315946	-1.947340	-1.000862	H	3.650420	0.698189	0.108843
C	1.155714	0.124802	1.100058	C	-4.324342	0.251252	-0.428964
C	-0.277852	0.642633	-1.648918	H	-4.893882	1.171390	-0.455037
C	-0.743879	-1.117544	2.139316	C	3.538958	2.827909	0.078108
H	-0.194233	-2.044216	2.056279	H	4.540420	2.952966	-0.311447
C	1.738106	1.391471	0.839481	C	0.828324	-4.129093	-2.011997
C	-2.156140	1.312508	2.340593	H	0.563673	-4.892686	-2.733277
H	-2.682618	2.254438	2.407553	C	0.333089	3.239457	-2.551989
C	-2.008658	-1.092834	2.666612	H	0.573998	4.240975	-2.887369
H	-2.471728	-2.008048	3.008653	C	3.799993	-2.497521	1.453391
C	0.027803	-2.977210	-1.886989	H	4.672457	-2.640689	2.077847
H	-0.845079	-2.889988	-2.524622	C	3.418435	-3.450680	0.544799
C	-2.972805	0.274991	-0.787025	H	3.992093	-4.363380	0.432285
C	-2.723985	0.120670	2.744933	C	2.732439	3.962342	0.304998
H	-3.732485	0.120736	3.137375	H	3.125506	4.949058	0.095459
C	-0.925989	2.977686	-2.041140	C	3.043940	-1.320536	1.573208
H	-1.684544	3.746575	-1.971381	H	3.337938	-0.572717	2.299044
C	-4.132799	-2.127132	-0.028689	C	-4.898150	-0.951281	-0.056279
H	-4.589657	-3.063000	0.268559	H	-5.946156	-0.980007	0.217238
C	0.986975	0.951379	-2.196586	B	-0.675769	-0.733174	-1.106599

Table S2. XYZ coordinates of the optimized geometry of **5˙**.

Atom	Coordinates			Atom	Coordinates		
	X	Y	Z		X	Y	Z
O	-2.537302	-0.784989	-1.503083	C	-1.709864	3.255998	-1.885603
O	-2.254965	0.740347	1.742787	H	-1.525678	4.319235	-1.977873
C	-1.840016	-0.562576	1.836928	C	-1.218032	-4.144001	-1.454658
C	2.680744	-0.542647	2.244740	H	-1.604209	-5.156664	-1.456150
H	2.196223	-0.705553	3.200370	C	0.419027	3.868827	1.304400
C	-1.336053	1.745376	1.591856	H	1.105490	4.699840	1.195722
C	-0.483104	-0.919481	1.661111	C	-0.656093	2.370803	-1.756629
C	-0.166563	-2.293438	1.754704	H	0.362360	2.738443	-1.740415
H	0.859353	-2.596900	1.600253	C	-1.594432	-1.776463	-1.471045
C	0.034178	1.463351	1.391907	C	0.165783	-3.920864	-1.437178
C	-0.864550	0.982545	-1.619311	H	0.849629	-4.761196	-1.428663
C	1.912910	-0.189833	1.151598	C	1.781025	0.283929	-1.302154
C	-2.825029	-1.507719	2.086432	C	-2.196442	0.535094	-1.623104
H	-3.848849	-1.175185	2.201236	C	-3.275914	1.416297	-1.733781
C	-1.825084	3.042560	1.634556	H	-4.284208	1.022224	-1.715662
H	-2.887523	3.193987	1.775377	B	0.250274	-0.045070	-1.396460
C	0.900214	2.571834	1.257094	C	-3.025621	2.771839	-1.866410
H	1.956293	2.389709	1.112135	H	-3.857680	3.461123	-1.952031
C	-0.216629	-1.509479	-1.431779	C	0.647688	-2.624240	-1.419451
C	4.719571	-0.332046	0.973421	H	1.716271	-2.444824	-1.392431
H	5.801753	-0.355190	0.906338	C	-2.101689	-3.078208	-1.469256
C	-2.477168	-2.851321	2.172421	H	-3.173814	-3.227857	-1.481391
H	-3.243268	-3.593449	2.359723	C	-0.946517	4.110133	1.488306
C	-1.142928	-3.239135	2.006140	H	-1.324404	5.124582	1.517032
H	-0.874669	-4.287134	2.054425	C	4.612849	0.357803	-1.400465
C	2.542993	0.036691	-0.114722	H	5.696680	0.355546	-1.438855
C	0.469185	0.097675	1.337993	C	3.871588	0.684889	-2.507525
C	3.972384	0.020341	-0.179547	H	4.364073	0.956041	-3.434437
C	4.085495	-0.636172	2.152595	C	2.459873	0.630291	-2.457377
H	4.660343	-0.911118	3.029109	H	1.899933	0.824913	-3.366405

Table S3. XYZ coordinates of the optimized geometry of [K(18-c-6)][**5**].

Atom	Coordinates			Atom	Coordinates		
	X	Y	Z		X	Y	Z
O	-2.575242	0.794623	-1.371217	C	-1.062241	3.037654	2.493573
O	-2.271076	-0.826419	1.608164	H	-0.754462	4.052462	2.718242
C	-0.272504	1.578739	-1.222972	C	-2.175749	3.099526	-1.511615
C	2.567183	0.289369	2.229609	H	-3.249139	3.217215	-1.607808
H	2.041858	0.362321	3.175542	C	4.013191	0.045008	-0.200855
C	-1.643379	1.817708	-1.370204	C	1.777970	-0.126581	-1.316288
C	-1.322222	-1.825281	1.512120	C	3.983779	0.359070	2.205684
C	0.010724	-1.531401	1.203110	H	4.517966	0.501665	3.139359
C	-1.822065	0.449404	1.890404	B	0.228808	0.080508	-0.915859
C	0.471663	-3.921268	1.354651	C	-0.593238	-2.249737	-1.905719
H	1.185263	-4.734946	1.290509	H	0.425657	-2.611881	-1.823252
C	-0.853534	-0.922642	-1.536090	C	0.385759	-0.111399	0.854122
C	-0.866858	-4.182983	1.636998	C	-1.592768	-3.117507	-2.340470
H	-1.210363	-5.198652	1.795264	H	-1.352176	-4.142593	-2.601223
C	0.898480	-2.610648	1.147367	C	4.695726	0.230551	1.030839
H	1.938275	-2.418022	0.916332	H	5.780403	0.270098	1.040959
C	-0.510219	0.837425	1.598175	C	0.057298	3.999364	-1.430304
C	1.874085	0.113801	1.052143	H	0.732862	4.847789	-1.457692
C	-1.767191	-3.123978	1.724571	C	-3.205776	-1.344708	-2.091413
H	-2.813668	-3.284386	1.955147	H	-4.215540	-0.956236	-2.159955
C	0.557077	2.707444	-1.279334	C	-2.910406	-2.664043	-2.419988
H	1.628296	2.562291	-1.181842	H	-3.703123	-3.328206	-2.746864
C	-2.747246	1.320048	2.454849	C	-1.320286	4.196574	-1.535110
H	-3.750127	0.959885	2.650664	H	-1.727754	5.195594	-1.644791
C	2.594069	0.013881	-0.162448	C	4.626577	-0.116963	-1.471590
C	-2.179929	-0.498358	-1.669026	H	5.709057	-0.107803	-1.549735
C	-2.369269	2.627575	2.748935	C	3.843670	-0.289298	-2.594690
H	-3.088918	3.313485	3.180604	H	4.324159	-0.417962	-3.559922
C	-0.151289	2.148224	1.928210	C	2.425122	-0.286564	-2.525524
H	0.854867	2.478908	1.708025	H	1.855877	-0.397253	-3.444173

C⋯B

$\rho(r)$: 0.0157 e/bohr^3

H(r): +0.00004 E_h/bohr^3

$\nabla^2\rho(r)$: +0.0347 e/bohr^5

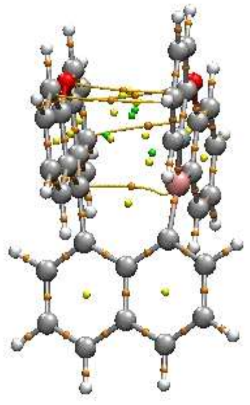

5$^{\bullet}$

C⋯B

$\rho(r)$: 0.1164 e/bohr^3

H(r): -0.09728 E_h/bohr^3

$\nabla^2\rho(r)$: -0.1269 e/bohr^5

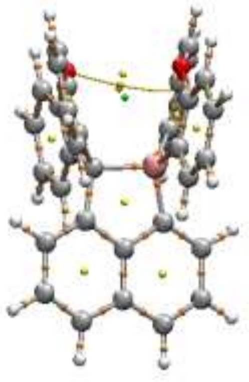

[K(18-c-6)][**5**]

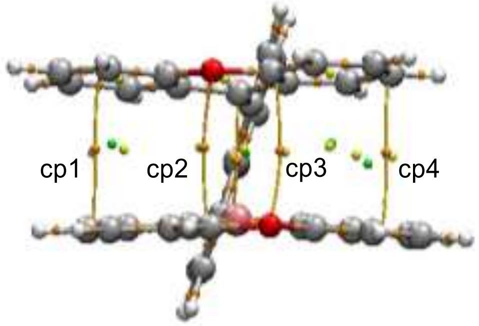

	cp1	cp2	cp3	cp4
$\rho(r)$ (e/bohr^3):	0.0056	0.0047	0.0044	0.0051
H(r) (E_h/bohr^3):	+0.00058	+0.00067	+0.00065	+0.00051
$\nabla^2\rho(r)$ (e/bohr^5):	+0.0145	+0.0163	+0.0154	+0.0130

Figure S26. AIM output for compound **5**$^{\bullet}$ and [K(18-c-6)][**5**] with relevant bond paths. The electron density (ρ(r)), energy density (H(r)) and Laplacian values ($\nabla^2\rho$(r)) at the given bond critical point of the central C-B contact are given. The inset depicts the values of ρ(r), H(r) and $\nabla^2\rho$(r) for the additional bond critical points of **5**$^{\bullet}$.

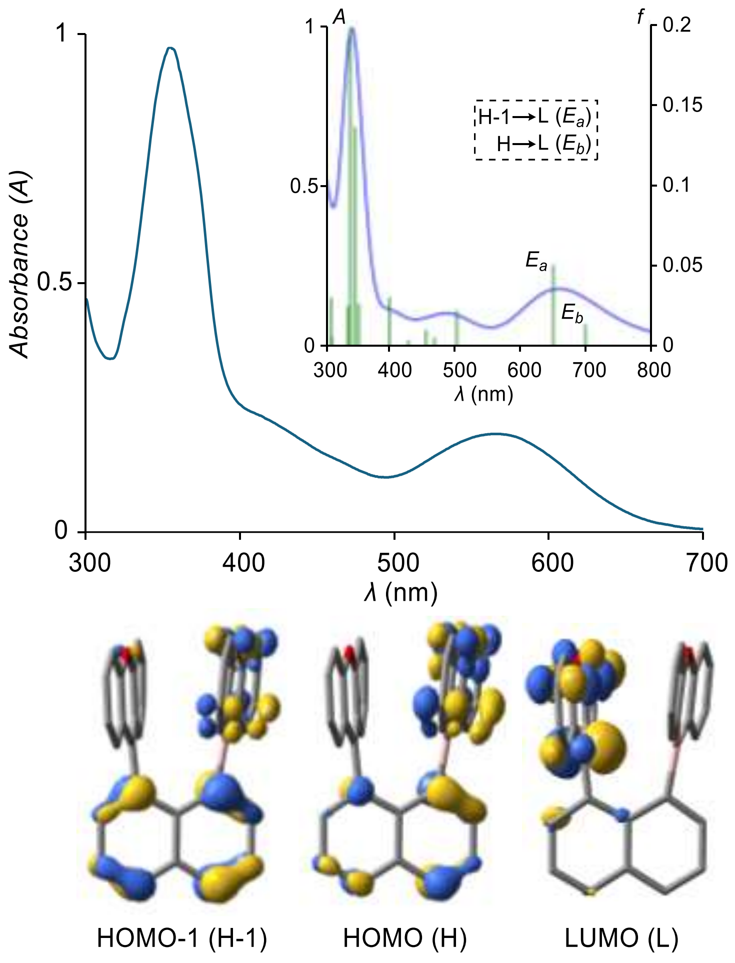

Figure S27. Normalized experimental and TD-DFT UV-vis spectrum with calculated vertical transitions (green bars) of [**5**][BF_4]. Bottom: HOMO-1, HOMO and LUMO of [**5**][BF_4] (0.05 isosurface value).

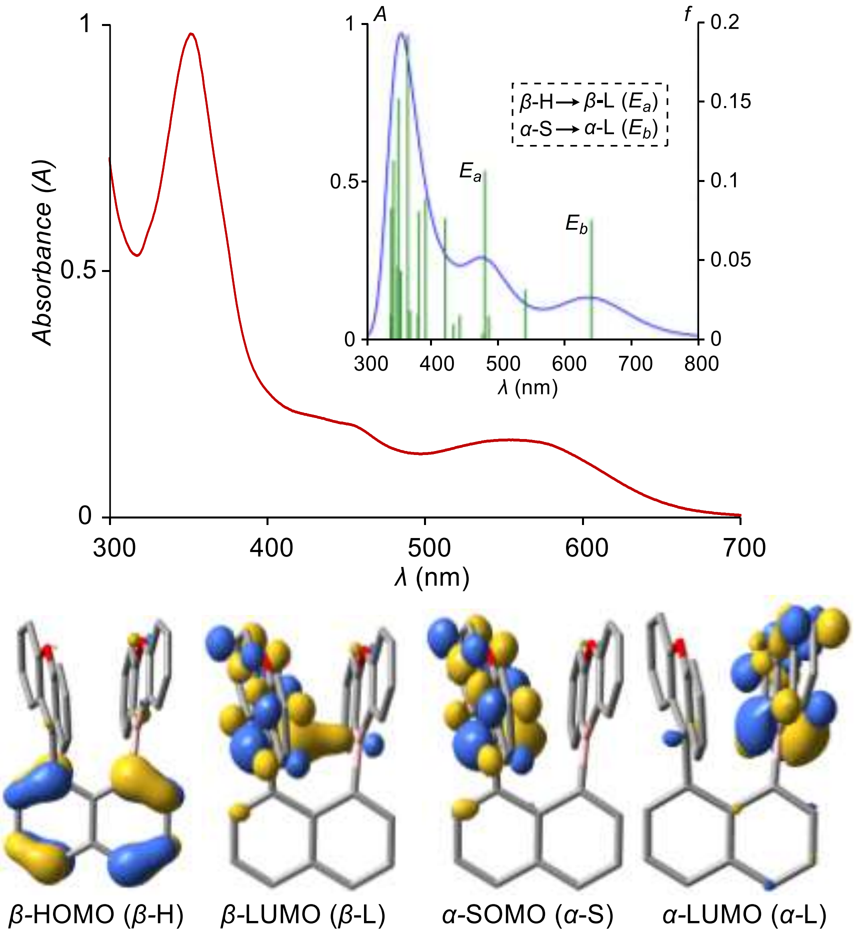

Figure S28. Normalized experimental and TD-DFT UV-vis spectrum with calculated vertical transitions (green bars) of **5**$^{\bullet}$. Bottom: β-HOMO (β-H), β-LUMO (β-L), α-SOMO (α-S) and α-LUMO (α-L) of **5**$^{\bullet}$ (0.05 isosurface value).

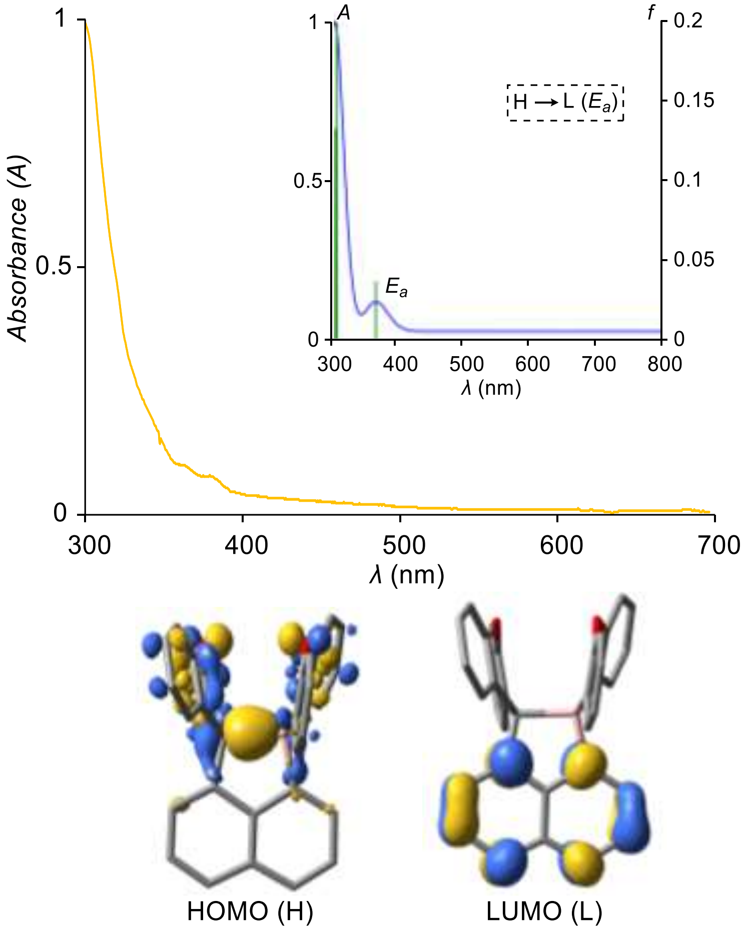

Figure S29. Normalized experimental and TD-DFT UV-vis spectrum with the calculated vertical transition (green bars) of [K(18-c-6)][**5**] Bottom: HOMO and LUMO of [K(18-c-6)][**5**] (0.05 isosurface value).

References

1. Melaïmi, M.; Sole, S.; Chiu, C.-W.; Wang, H.; Gabbaï, F. P. Structural and Electrochemical Investigations of the High Fluoride Affinity of Sterically Hindered 1,8-Bis(boryl)naphthalenes. *Inorg. Chem.* **2006**, *45*, 8136-8143.
2. Rigaku Oxford Diffraction, (2022), *CrysAlisPro Software system*, version 171.42.61a, Rigaku Corporation, Wroclaw, Poland.
3. Sheldrick, G. M. *SADABS, Version 2007/4, Bruker Analytical X-ray Systems, Inc., Madison, Wisconsin, USA*, 2007.
4. Sheldrick, G. M. SHELXT - integrated space-group and crystal-structure determination. *Acta Crystallogr. A* **2015**, *71*, 3-8.
5. Sheldrick, G. M. *SHELXL-2014: Program for Crystal Structure Refinement*, University of Göttingen, Germany, 2014.
6. Dolomanov, O. V.; Bourhis, L. J.; Gildea, R. J.; Howard, J. A. K.; Puschmann, H. OLEX2: a complete structure solution, refinement and analysis program. *J. Appl. Crystallogr.* **2009**, *42*, 339-341.
7. Stoll, S.; Schweiger, A. EasySpin, a comprehensive software package for spectral simulation and analysis in EPR. *J. Magn. Reson.* **2006**, *178*, 42-55.
8. Frisch, M. J.; Trucks, G. W.; Schlegel, H. B.; Scuseria, G. E.; Robb, M. A.; Cheeseman, J. R.; Scalmani, G.; Barone, V.; Petersson, G. A.; Nakatsuji, H.; Li, X.; Caricato, M.; Marenich, A. V.; Bloino, J.; Janesko, B. G.; Gomperts, R.; Mennucci, B.; Hratchian, H. P.; Ortiz, J. V.; Izmaylov, A. F.; Sonnenberg, J. L.; Williams; Ding, F.; Lipparini, F.; Egidi, F.; Goings, J.; Peng, B.; Petrone, A.; Henderson, T.; Ranasinghe,

D.; Zakrzewski, V. G.; Gao, J.; Rega, N.; Zheng, G.; Liang, W.; Hada, M.; Ehara, M.; Toyota, K.; Fukuda, R.; Hasegawa, J.; Ishida, M.; Nakajima, T.; Honda, Y.; Kitao, O.; Nakai, H.; Vreven, T.; Throssell, K.; Montgomery Jr., J. A.; Peralta, J. E.; Ogliaro, F.; Bearpark, M. J.; Heyd, J. J.; Brothers, E. N.; Kudin, K. N.; Staroverov, V. N.; Keith, T. A.; Kobayashi, R.; Normand, J.; Raghavachari, K.; Rendell, A. P.; Burant, J. C.; Iyengar, S. S.; Tomasi, J.; Cossi, M.; Millam, J. M.; Klene, M.; Adamo, C.; Cammi, R.; Ochterski, J. W.; Martin, R. L.; Morokuma, K.; Farkas, O.; Foresman, J. B.; Fox, D. J. *Gaussian 16 Rev. C.01*, Wallingford, CT, 2016.
9. Glendening, E.D.; Badenhoop, J. K.; Reed, A. E.; Carpenter, J. E.; Bohmann, J. A.; Morales, C. M.; Karafiloglou, P.; Landis, C. R.; Weinhold, F. *NBO 7.0, Theoretical Chemistry Institute*, University of Wisconsin, Madison, WI, 2018.
10. Hanwell, M. D.; Curtis, D. E.; Lonie, D. C.; Vandermeersch, T.; Zurek, E.; Hutchison, G. R. Avogadro: an advanced semantic chemical editor, visualization, and analysis platform. *J. Cheminformatics* **2012**, *4*, 17.
11. Lu, T.; Chen, F. Multiwfn: A multifunctional wavefunction analyzer. *J. Comput. Chem.* **2012**, *33*, 580-592.
12. Humphrey, W.; Dalke, A.; Schulten, K. VMD: Visual molecular dynamics. *J. Mol. Graph.* **1996**, *14*, 33-38.
